# Morphometric analysis of sella turcica in growing patients: an observational study on shape and dimensions in different sagittal craniofacial patterns

**DOI:** 10.1038/s41598-019-55916-y

**Published:** 2019-12-17

**Authors:** Michele Tepedino, Michele Laurenziello, Laura Guida, Graziano Montaruli, Giuseppe Troiano, Claudio Chimenti, Marco Colonna, Domenico Ciavarella

**Affiliations:** 1 Department of Biotechnological and Applied Clinical Sciences, University of L’Aquila, L’Aquila, Italy; 2 Department of Clinical and Experimental Medicine, University of Foggia, Foggia, Italy

**Keywords:** Anatomy, Dentistry, Medical imaging, Paediatrics

## Abstract

The aim of this study was to evaluate the differences in sella dimensions and shape between growing patients with Class I, Class II, and Class III skeletal malocclusions, evaluated through morphometric analysis. Seventy-eight subjects aged between 9 and 13 years were selected and assigned to either the Class I, Class II, or Class III groups according to the measured ANB angle (the angle between the Nasion, skeletal A-point and skeletal B-point). Six landmarks were digitised to outline the shape of the sella turcica. Linear measurements of the sella length and depth were also performed. Procrustes superimposition, principal component analysis, and canonical variate analysis were used to evaluate the differences in sella shape between the three groups. A one-way MANOVA and Tukey’s or Games-Howell tests were used to evaluate the presence of differences in sella dimensions between the three groups, gender, and age. The canonical variate analysis revealed a statistically significant difference in sella shape between the Class I and the Class II groups, mostly explained by the CV1 axis and related to the posterior clinoidal process and the floor of the sella. No differences were found regarding linear measurements, except between subjects with different age. These differences in sella shape, that are present in the earlier developmental stages, could be used as a predictor of facial growth, but further studies are needed.

## Introduction

The sella turcica is an important structure within the craniofacial complex. It acts as a radiographic landmark, which is useful for orthodontic diagnosis of maxillofacial disharmonies, for the assessment of growth through superimposed tracings, and for the assessment of the results of orthodontic treatment^1–3^. The sella houses the pituitary gland which produces fundamental hormones like prolactin, growth hormones, thyroid-stimulating hormones, and follicular-stimulating hormones, among others^4^. Systemic pathologies and alterations in the function of the pituitary gland may result in morphological alterations of the sella turcica, or vice versa, morphological anomalies of the sella could reveal undetected pathologies^5^. Alterations in the size of the sella are frequently caused by pathologies: enlargement is usually caused by adenomas, empty sella syndrome, cysts, or aneurysms, while an abnormally small sella can be observed in primary hypopituitarism, or patients with a growth hormone deficiency or with other syndromes^6^. The shape of the sella can be also altered by conditions like Down syndrome, Williams syndrome, Seckel syndrome, and lumbosacral myelomeningocele^6,7^. Indeed, pituitary gland development ceases before the calcification of the sella turcica, therefore the development of the sella is strongly influenced by that of the pituitary gland^8^. In addition, the anterior and posterior sections of the pituitary gland and the sella turcica have different embryological origins, with the anterior part developing from the cells of the neural crest and the posterior part developing from the para-axial mesoderm, which is highly dependent on notochordal induction^9,10^. This close relationship, together with the differences in embryogenesis, explain the observation that anomalies of the anterior wall of the sella turcica appear to be associated with alterations of the frontonasal area and defects in the body axis, while anomalies of the posterior wall of the sella appear to be associated with brain alterations^8,11^.

Some authors have investigated whether craniofacial growth disharmonies, like those observed in skeletal malocclusions, can be associated with morphological anomalies of the sella turcica. Dasgupta *et al*.^12^ and Meyer-Marcotty *et al*.^13^ found an association between sella bridging (i.e., abnormal calcification of the dura mater between the clinoidal processes) and skeletal Class II and Class III malocclusions, respectively, while Alkofide *et al*.^1^ found differences in the sella diameter between Class II and Class III subjects. However, these results were based on a subjective assessment or linear measurements, both of which provide limited information about the shape and morphology of the sella turcica. Geometric morphometric analysis has been used several times in the orthodontic field to provide a quantitative and objective evaluation of the shape and morphology^14–18^. Andredaki *et al*.^6^ used geometric morphometric analysis to provide normative data on the sella shape.

The orthopaedic treatment of sagittal skeletal malocclusions is performed in growing subjects, so it is useful to investigate the morphology of the sella turcica in these kinds of patients to retrieve information on their craniofacial development. It is known from longitudinal studies that the growth of the sella turcica decreases rapidly after the first year of life, increases during puberty, and slows down and ceases in late adolescence^19^. The shape of the sella turcica, however, is established in the early embryonic structure^7^. If the sella turcica has a different shape and dimensions in subjects with different skeletal malocclusions, this could be used as an early predictor of the future growth of the craniofacial complex.

The aim of the present study was to combine geometric morphometric analysis and traditional morphometrics (linear measurements), to compare the sella shape and dimensions in growing patients with Class I, Class II, and Class III skeletal malocclusions. The null hypothesis was that no differences in the sella shape and/or dimensions exist between subjects with Class I, Class II, or Class III malocclusions.

## Materials and Methods

This study was conducted following the STROBE guidelines for observational studies. The records of patients referred to the Dental Clinic of the University of L’Aquila between January 2012 and January 2018 were screened by a blinded operator for the following criteria: aged between 9 and 13 years; presence of Class I skeletal malocclusion with an ANB angle (the angle between Nasion, skeletal A-point and skeletal B-point) between 1° and 3°, or Class II skeletal malocclusion with an ANB angle greater than 5°, or Class III skeletal malocclusion with an ANB angle less than −1°; and availability of good quality lateral cephalograms. The procedures that were followed were in accordance with the Helsinki Declaration of 1975 and subsequent revisions. All possible attempts were made to contact the patients whose records were selected for inclusion in the study sample, in order to obtain their written informed consent. If this was not achieved after all possible attempts had been made, the need for consent was waived by the Ethical Committee. All the procedures that were followed were approved by the Ethical Committee of the University of L’Aquila (protocol no. 54461, ID 42/2018).

The sample size calculation (G*Power version 3.1.9.2, Franz Faul, Universität Kiel, Germany), performed for a one-way ANOVA for linear measurements with an effect size f of 0.38, was calculated from the data of a previous study by Alkofide^1^. This calculation revealed that a total sample size of 72 subjects was needed to be able to reject the null hypothesis with 80% power and a type I error probability of 0.05.

The records of 250 patients were screened for the eligibility criteria: 147 patients were excluded due to age, and 25 patients out of the 103 that met the age criteria were excluded because they showed an ANB angle that was not within the selected range. Therefore, 78 patients were included in the study. Lateral cephalograms for the selected patients were collected and depending on the ANB angle the patients were allocated to either the Class I, Class II, or Class III groups. The radiographs were anonymised by assigning a numerical code. The Class I group was comprised of 12 females (mean age 10.8 ± 1.8) and 13 males (mean age 10.2 ± 1.8), the Class II group was comprised of 24 females (mean age 10.5 ± 2.4) and 10 males (mean age 10.2 ± 2.1), and the Class III group was comprised of 9 females (mean age 10.0 ± 2.4) and 10 males (mean age 9.3 ± 2.7).

### Geometric morphometric analysis

**Figure 1:**
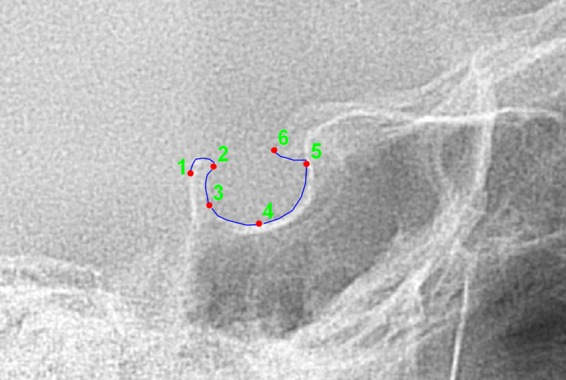
Morphometric evaluation of the sella turcica. Red dots are the digitised landmarks: 1, the most posterior point of the posterior clinoidal process; 2, the dorsum sellae point; 3, the most posterior point of the posterior wall of the sella turcica; 4, the deepest point (sella floor) of the sella; 5, the most anterior point of the anterior wall of the sella; 6, the most posterior point of the anterior clinoidal process.

A comprehensive dataset of the lateral cephalograms for the whole sample was created with tpsUtil software (tps Utility Program version 1.76, Morphometrics at SUNY Stony Brook. http://life.bio.sunysb.edu/morph/). The dataset was imported into tpsDig2 software (tpsDig 2 version 2.31, Morphometrics at SUNY Stony Brook. http://life.bio.sunysb.edu/morph/) where the images were first calibrated using the ruler positioned over the craniostat to scale the image to the real dimensions, then the following six landmarks were positioned by a single operator in a single-blind fashion using the Frankfurt plane as the reference plane (Fig. 1): (1) the most posterior point of the posterior clinoidal process, (2) the dorsum sellae point, (3) the most posterior point of the posterior wall of the sella turcica, (4) the deepest point (sella floor) of the sella, (5) the most anterior point of the anterior wall of the sella, and (6) the most posterior point of the anterior clinoidal process. The created.TPS files were imported into MorphoJ software (MorphoJ version 1.06d, http://www.flywings.org.uk/morphoj_page.htm)^20^. A second operator assigned each subject to the corresponding group using the “edit classifiers” function. Then, all tracings were registered on the six landmarks using Procrustes superimposition to calculate the average shape and the centroid size.

To check for the presence of outliers, a diagram was plotted with the cumulative distribution of the Mahalanobis distances of individual specimens from the average shape of the entire sample, to test the presence of a multivariate normal distribution of the data. To calculate the error in the landmark digitisation, 10 specimens were randomly selected, and landmarks were digitised twice, with a one-day interval between each one. A Procrustes fit and a principal component analysis (PCA) was obtained from the repeated measurements, and the total variance of the PCA was compared with the total variance of the sample^21^.

### Size measurements

**Figure 2:**
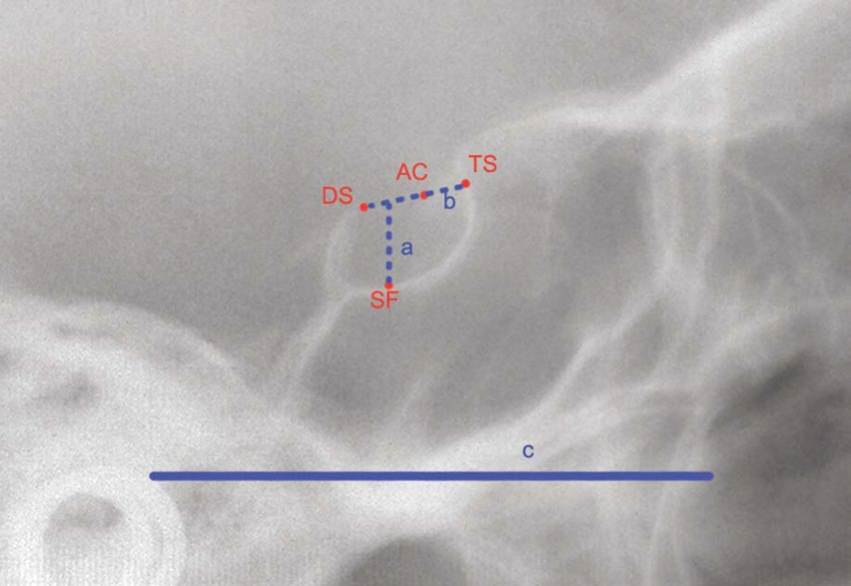
Schematic representation of the linear measurements of sella dimensions. *DS*, dorsum sellae; *AC*, anterior clinoidal process; *TS*, tuberculum sellae; *SF*, sella floor; *a*, measurement of sella depth; *b*, measurement of sella length; *c*, Frankfurt plane used as a reference for tracing the *a* line.

The sella dimensions were calculated with ImageJ software (ImageJ version 1.5, National Institute of Health, USA) used by a single operator in a single-blind fashion. The anonymised lateral cephalograms were imported into the software, calibrated using the ruler that had been positioned onto the craniostat in each radiograph, then the following measurements were taken (Fig. 2): sella length, measured as the distance between the dorsum sellae and the tuberculum sellae; and sella depth, measured as the distance, perpendicular to the Frankfurt plane, between the line connecting the dorsum sellae and the tuberculum sellae, and the deepest point of the sella floor.

To calculate the error of this method, 20 subjects were randomly selected (www.randomizer.org) from the whole sample, and the same operator repeated the sella measurements after a two-week interval. An intraclass correlation coefficient (ICC) was calculated between the two sets of measurements to evaluate the intraoperator reliability.

### Statistical analysis

For the geometric morphometric analysis, a covariance matrix was calculated from the Procrustes fit of the whole sample.

To determine if subjects in different gender and age categories could be pooled, a preliminary test was performed. A canonical variate analysis (CVA) was performed to compute Mahalanobis distances (with 10,000 permutation rounds) to investigate the degree of dissimilarity in the sella shape between males and females, and to compute Procrustes distances (with 10,000 permutation rounds) between different age categories (category 1, between 9 and 10 years of age; category 2, between 11 and 12 years of age; category 3, 13 years of age).

To compare the geometric morphometric properties of the sella turcica between the three groups of skeletal malocclusions, a CVA was performed and Mahalanobis distances (with 10,000 permutation rounds) were calculated to investigate the degree of dissimilarity in the sella shape between subjects with a skeletal Class I, Class II, or Class III malocclusion^22^. In addition, a discriminant function analysis (DFA) with leave-one-out cross-validation was performed to evaluate how the subjects can be correctly classified into the skeletal malocclusion categories based on the shape of their sella (Mahalanobis distances in a permutation test with 10,000 rounds).

Regarding size measurements, after calculating descriptive statistics, a possible effect of gender and age was also evaluated to confirm that all the subjects could be pooled. A one-way MANOVA was performed to evaluate the presence of differences in sella length and sella depth between the three groups and between different age categories. A pairwise comparison using the Tukey’s honestly significant difference (HSD) test or the Games-Howell post-hoc test was performed depending on the results of the homogeneity of variances test. To also evaluate the effect of gender, after evaluating the data distribution with a Shapiro-Wilk normality test, an Independent samples T-test was performed to evaluate the presence of differences between linear measurements (sella depth and sella length) across gender.

For all the statistical tests performed, the level of significance was set at 0.016 after applying the Bonferroni correction (0.05/3 = 0.016). Procrustes superimpositions, CVA, and DFA were performed using the MorphoJ software (MorphoJ version 1.06d, http://www.flywings.org.uk/morphoj_page.htm)^20^, while the other statistical tests were performed using the SPSS software (SPSS Inc. Released 2004. SPSS for Windows, Version 13.0. Chicago, SPSS Inc.).

## Results

Regarding the error of the method used for the linear measurements, the calculated ICC coefficient was excellent (>0.85) for both variables, revealing good intraobserver reliability of the measurements. Regarding the error of the method for digitised landmarks, the total variance of the repeated measurements calculated from the PCA (0.0018) was considered negligible when compared with the total variance of the sample (0.031 for the Class I group, 0.028 for the Class II group, 0.034 for the Class III group, and 0.031 for the whole sample).

### Geometric morphometric analysis

**Figure 3:**
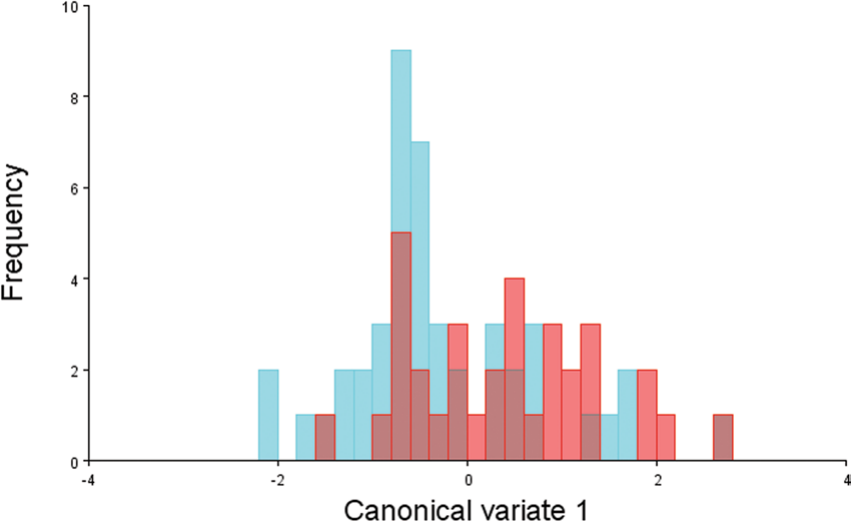
Scores from the canonical variate analysis of gender effect on sella shape; red dots = female subjects; light blue dots = male subjects.

**Figure 4:**
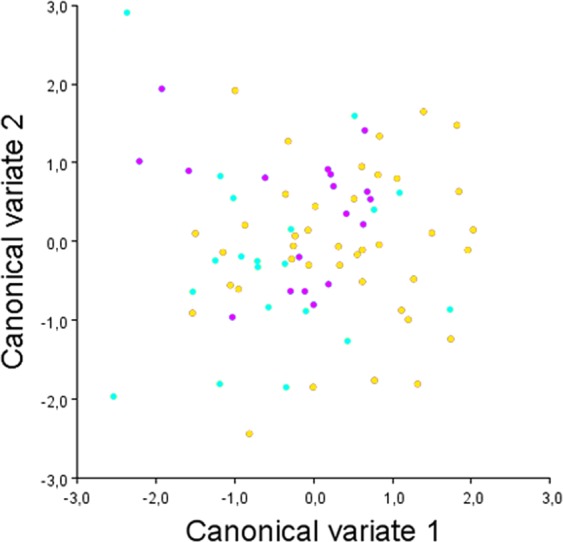
Results from the canonical variate analysis. A scatterplot of the canonical variate (CV) 1 against the CV2, plotted according to the following age categories: 9 to 10 years of age (yellow dots), 11 to 12 years of age (turquoise dots), and 13 years of age (purple dots).

The CVA with permutation tests of the sella turcica shape pooled by gender and age revealed no significant differences between males and females (Mahalanobis distance among groups = 0.68, *p* = 0.356) (Fig. 3) and no difference between age categories (Fig. 4, Table 1).

**Table 1:** Procrustes distances between different gender and age categories.

		Females			Males	
		9–10 years of age (n = 16)	11–12 years of age (n = 9)	13 years of age (n = 8)	9–10 years of age (n = 24)	11–12 years of age (n = 11)
Females	11–12 years of age (n = 9)	0.09 (0.108)	—	—	—	—
	13 years of age (n = 8)	0.07 (0.374)	0.07 (0.614)	—	—	—
Males	9–10 years of age (n = 24)	0.05 (0.484)	0.09 (0.123)	0.04 (0.932)	—	—
	11–12 years of age (n = 11)	0.08 (0.253)	0.12 (0.091)	0.08 (0.495)	0.07 (0.340)	—
	13 years of age (n = 10)	0.05 (0.718)	0.10 (0.221)	0.11 (0.286)	0.08 (0.160)	0.07 (0.587)

Procrustes distances (p value); p value from permutation test with 10,000 permutation rounds; *statistically significant with p < 0.05; **statistically significant with p < 0.01.

**Figure 5:**
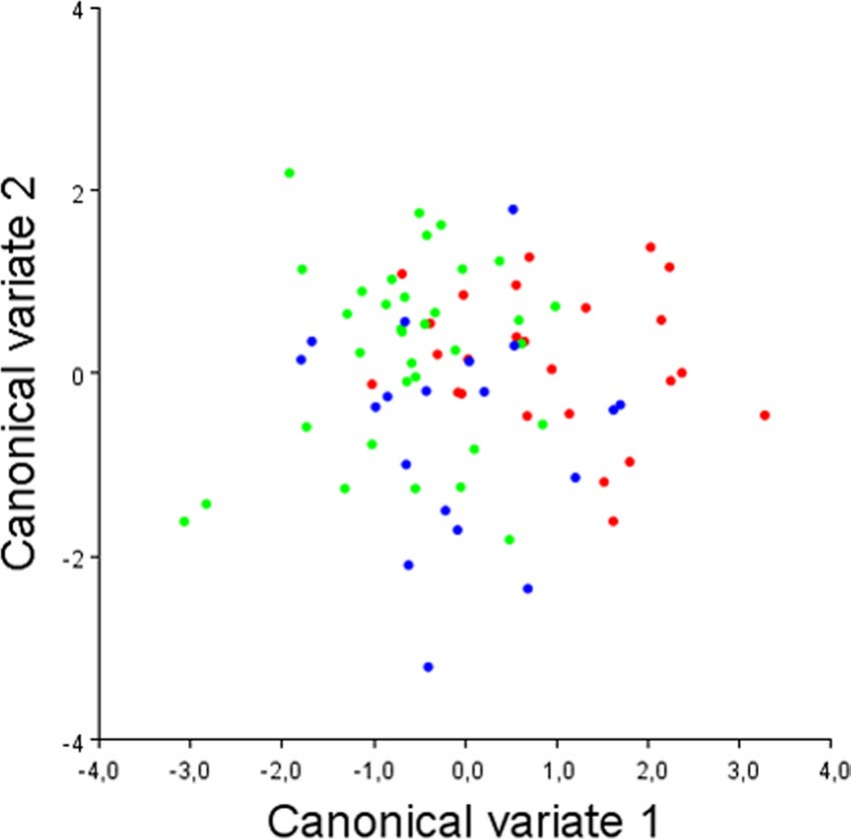
Results from the canonical variate analysis. A scatterplot of the canonical variate (CV) 1 against the CV2, plotted according to the Class I (red dots), the Class II (green dots), and the Class III (blue dots) groups.

**Figure 6:**
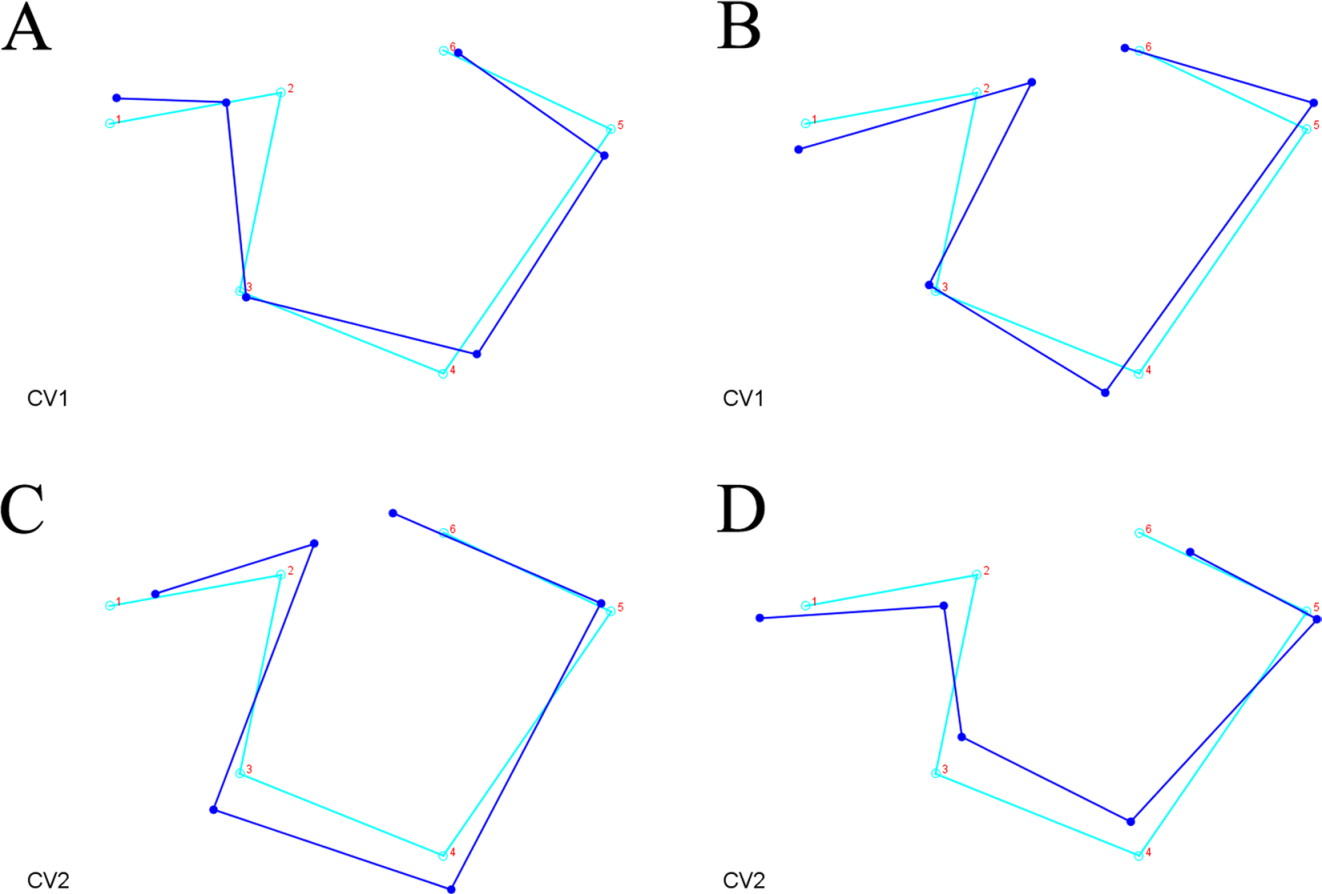
Wireframe graphs from the canonical variate analysis (CVA) of the skeletal Class I, skeletal Class II, and skeletal Class III groups. The starting shape is outlined in light blue, while the target shape is outlined in dark blue. (**A**) Wireframe graph of canonical variate 1 (CV1) showing the target shape change towards the negative end of the axis. (**B**) Wireframe graph of CV1 showing the target shape change towards the positive end of the axis. (**C**) Wireframe graph of CV2 showing the target shape change towards the negative end of the axis. (**D**) Wireframe graph of CV2 showing the target shape change towards the positive end of the axis.

Regarding the CVA between skeletal classes, Mahalanobis distances were reported due to sample size and because isotropy is not required, since the shape variation was not expected to be similar in all the space directions^22–25^. Results from the CVA revealed a statistically significant difference for the Mahalanobis distance between the Class I group and the Class II group (Table 2, Fig. 5). The Mahalanobis distance between the Class I group and the Class III group was 1.28 but did not reach statistical significance (*p* = 0.027), and a smaller and non-significant Mahalanobis distance was found between the Class II and the Class III group. The CVA plot (Fig. 5) shows that most of the variation of the Class I group and the Class II group was along the CV1 axis (Fig. 6A), that is the axis that best describes the shape difference between those two groups, while the variation of the Class III group was mainly related to the CV2 axis (Fig. 6B). Comparing the Class I and the Class II groups, shape changes for the Class I group along the CV1 axis were found with landmarks 1, 2, 4, and 5 in the positive direction, while shape changes for the Class II group along the same axis were found with the same landmarks, but with changes in an opposite direction. Those landmarks are associated with the posterior clinoidal processes, the floor of the sella, and the concavity of the anterior wall of the sella. The Class I group was mainly distributed towards the positive end of the CV1 axis, showing the posterior interclinoidal processes located closer to the anterior clinoidal processes, an anterior wall of the sella that is markedly inclined towards the anterior, and in general a narrower and deeper shape of the sella turcica with the floor displaced inferiorly (Fig. 6B). In contrast, the Class II group was mainly distributed towards the negative end of the CV1 axis, showing a wider interclinoidal aperture with a posteriorly positioned posterior clinoidal processes, a concave anterior wall, and a wide floor of the sella (Fig. 6A). The anterior clinoidal processes and the posterior wall of the sella (landmarks 6 and 3, respectively) showed less variation in these two groups, compared with the other structures. Shape changes for the Class III group along the CV2 axis were found with landmarks 1, 2, 3, 4, and 6. Those landmarks are associated with the anterior and posterior clinoidal processes, and the floor and the posterior wall of the sella, and showed changes in the negative direction of the CV2 axis (Fig. 6C): a sella floor displaced inferiorly, a concave posterior wall, and the anterior and posterior clinoidal processes that tend to be close one to another. Together, the first two CV axes accounted for 100% of the total variation (CV1 = 79.3%, CV2 = 20.7%), but the scatter plot from CV1 and CV2 (Fig. 5) showed that the three groups were overlapping and not clearly clustered.

**Table 2:** Canonical variate analysis (CVA) between the three groups.

	Mahalanobis distances (p value)	
	Class I group	Class II group
Class II group	1.56** (<0.001)	—
Class III group	1.28 (0.027)	0.98 (0.169)

p value from permutation test with 10,000 permutation rounds; *statistically significant with p < 0.016.

**Figure 7:**
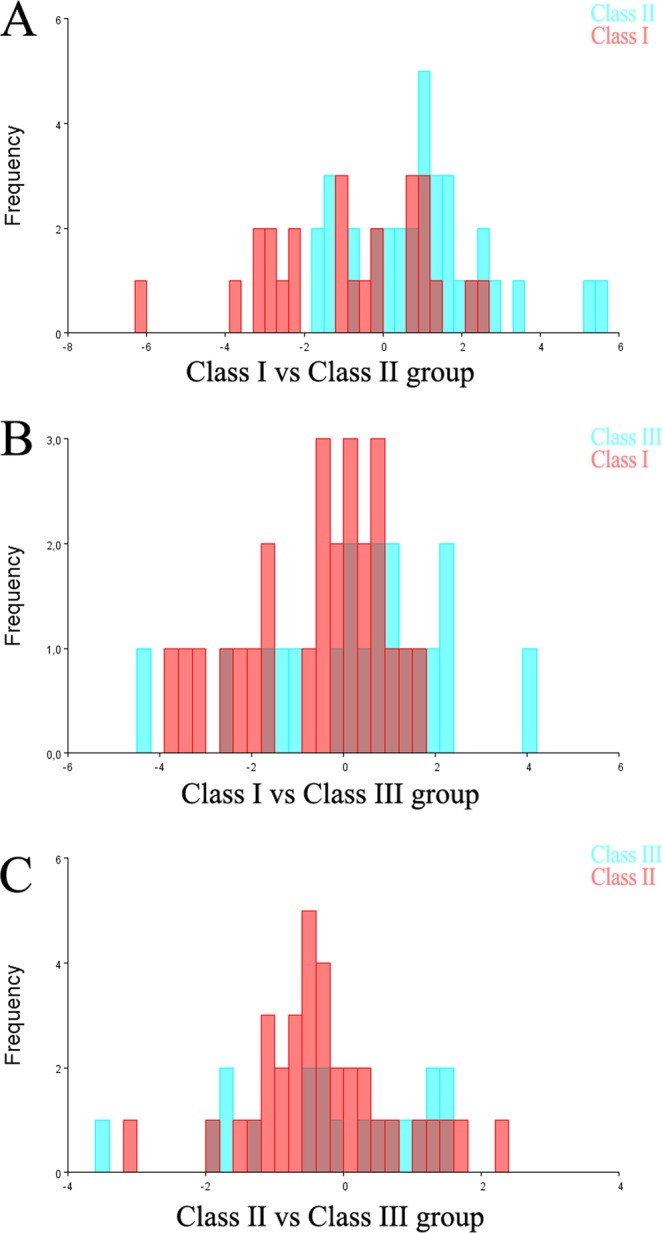
Discriminant function analysis (DFA) with leave-one-out cross-validation. (**A**) Class I group (red colour) vs. Class II group (light blue colour) scores. (**B**) Class I group (red colour) vs. Class III group (light blue colour) scores. (**C**) Class II group (red colour) vs. Class III group (light blue colour) scores.

The pairwise DFA with cross-validation between the Class I group and Class II group was able to correctly classify 64% of the Class I subjects and 73.5% of the Class II subjects. The pairwise DFA with cross-validation between the Class I group and Class III group was able to correctly classify 56% of the Class I subjects and 68% of the Class III subjects. The pairwise DFA with cross-validation between Class II group and Class III group was able to correctly classify 67% of the Class II subjects and 47% of the Class III subjects (Fig. 7, Table 3).

**Table 3:** Classification/misclassification tables from the discriminant function analysis with cross-validation of the skeletal malocclusion groups.

True	Allocated to		Total
	Class I group	Class II group	
**Class I vs. Class II group**			
Class I group	16	9	25
Class II group	9	25	34
**True**	**Allocated to**		**Total**
	**Class I group**	**Class III group**	
**Class I vs. Class III group**			
Class I group	14	11	25
Class III group	6	13	19
**True**	**Allocated to**		**Total**
	**Class II group**	**Class III group**	
**Class II vs. Class III group**			
Class II group	23	11	34
Class III group	10	9	19

### Size measurements

Descriptive statistics for the sella dimensions are reported in Table 4. The Independent samples T-test revealed no statistically significant difference for the sella depth (mean difference = 0.49, p = 0.137, 95% CI [−0.16, 1.13], assuming equal variances) and sella length (mean difference = 0.06, p = 0.847, 95% CI [−0.57, 0.69], assuming equal variances) between genders. A statistically significant difference was found for the sella length and sella depth between subjects of 9–10 years of age and patients of 13 years of age (Tables 5 and 6), regardless of the skeletal class, with measurements increasing in older patients. No statistically significant differences between the three skeletal malocclusion groups were found for the sella length or sella depth (Table 5). The null hypothesis was partly rejected as there was a difference in the sella shape, but not the sella dimensions, between the three groups, and a difference in sella dimensions between the different age categories.

**Table 4:** Descriptive statistics for the linear measurements of the sella turcica in all three groups.

	Class I group (n = 25)	Class II group (n = 34)	Class III group (n = 19)
Sella length (mm)	8.3 ± 1.5	8.7 ± 1.5	8.6 ± 0.9
Sella depth (mm)	7.9 ± 1.3	8.4 ± 1.6	8.7 ± 1.1

Mean ± SD.

**Table 5:** Results of a one-way MANOVA for the linear measurements of the sella depth and sella length between age categories and different skeletal classes.

	Wilk’s Λ	F	p value	Partial η^2^
Intercept	0.02**	1601.92	<0.001	0.98
Age categories	0.68**	7.457	<0.001	0.33
Skeletal class	0.93	1.29	0.278	0.03

**Statistically significant for p < 0.01.

**Table 6:** Results of post-hoc test for age categories following a one-way MANOVA for the linear measurements of sella depth and sella length.

	(I)	(J)	Mean difference	p value	95% confidence interval	
					lower bound	upper bound
Sella length	1	2	−0.88	0.025	−1.67	−0.09
		3	−1.71*	<0.001	−2.51	−0.91
	2	3	−0.83	0.094	−1.76	0.11
Sella depth	1	2	−0.04	0.994	−0.94	0.85
		3	−1.11*	0.013	−2.02	−0.20
	2	3	−1.07	0.046	−2.13	0.01

*Statistically significant for p < 0.016. Category 1, between 9 and 10 years of age; Category 2, between 11 and 12 years of age; Category 3, 13 years of age.

## Discussion

The morphology of the sella turcica has been the subject of many studies, probably due to its clear appearance on lateral cephalograms and its importance as a cephalometric landmark for orthodontic and maxillofacial diagnosis. Several authors have attempted to categorise its shape and association with normal or abnormal conditions^1,5^. From assessing the lateral cephalograms of children aged 1–12 years, Gordon and Bell^26^ classified the sella shape as either circular, oval, or flattened. Some authors introduced the definitions of ‘J-shaped sella’ and ‘omega sella’^27^ but these were later revealed to be radiological misconceptions by other authors^28^. Other proposed anomalies of the sella shape include the flatness or concavity of the sella floor, the angulation of the contour of the tuberculum sellae, the shape of the anterior and posterior clinoidal processes, and the presence of the sella turcica bridge^29,30^. A comprehensive classification of the sella turcica shape was provided by Axelsson *et al*.^31^, who assessed the records of 72 Norwegian subjects from 6 to 21 years of age to provide normative values. These authors recognised the existence of six main sella types: normal sella turcica, oblique anterior wall, double-contoured sella, sella turcica bridge, notching of the posterior wall of the sella, and pyramidal shape of the dorsum sellae^31^. However, these alterations can be seen in both normal patients and subjects with pathological conditions^1,29^. In addition, these shape alterations are qualitative and rely on subjective assessments, and many authors have highlighted the difficulty in assigning some patients to a specific category^26,28^. The introduction of geometric morphometric analysis has represented a great advantage for scientific research, as it has enabled the objective and quantitative study of the pure shape properties of anatomical structures^14^. Only one study has used geometric morphometric analysis to provide normative values for sella shape in subjects of different gender and age^6^. These authors found no effect of patient age on the sella shape, and a limited effect of gender on the sella shape^6^. In the present study, no effect of gender on the sella shape was observed (Fig. 3).

The CVA revealed a highly significant difference in the sella shape between the Class I group and the Class II group (Table 2, Fig. 7). This difference was mainly explained by the CV1 axis (Fig. 5); the Class I group was mainly distributed towards the positive end of the CV1 axis, while the Class II group was mainly distributed towards the negative end of the CV1 axis (Fig. 6). Although these results were statistically significant, a certain overlap between the different skeletal patterns was observed in both the PCA and the CVA analyses (Fig. 5). Also, the DFA with cross-validation between the Class I and Class II groups offered a moderate (>70%) accuracy in classifying the Class II cases, and a slightly worse performance (64%) in correctly classifying the Class I cases. The Class III group showed less variation in the anterior wall of the sella (landmark 5), and a large variation in the anterior and posterior clinoidal processes, in the floor of the sella, and in the posterior wall of the sella, mostly explained by the CV2 axis in the negative direction (Fig. 6C). However, such shape variations were not statistically different from the Class I and the Class II group, and the pairwise DFA with cross-validation showed a moderate percentage (68%) of correctly classified Class III subjects against the Class I group and a low percentage (47%) of correctly classified Class III subjects against the Class II group.

These results suggest that patients with a disharmonic growth of the maxillofacial complex (i.e., with a skeletal Class II malocclusion) have a different morphology of the sella turcica compared with subjects with a normal growth (the Class I group), mostly concerning the anterior wall, the floor of the sella, and the anterior clinoidal processes, even if this difference could be difficult to recognise during a clinical examination. It is known that the sella turcica is formed at the most cranial end of the notochord, and its anterior and lower wall are located at a boundary area with different maxillofacial developmental fields (frontonasal field, maxillary field, palatal field, and mandibular field) that are formed by the migration of cells from the neural crest or the para-notochordal mesoderm, as demonstrated by many studies on genetic malformations^8,32^. It is interesting to note that the Class II group had shape changes that mostly involved the anterior wall and floor of the sella; this area is embryologically derived from the neural crest, similar to the fronto-maxillary complex which this area is strongly connected to and integrated with^33^. Some studies have demonstrated that when using points other than the cranial base as a reference for measurements, most Class II malocclusions are caused by a protrusive maxilla^34^. On the other hand, the shape variation in the Class III group, unlike the Class I and the Class II groups, involved the posterior wall of the sella, which shares a common origin with the posterior cranial base from the para-axial mesoderm. Some studies have revealed that variations in the length and inclination of the posterior cranial base are related to mandibular prognathism^33^. However, only the shape variations in the Class II group were statistically different from the norm (i.e., the Class I group), while the shape variations in the Class III group were not markedly distinct from those in the Class I group, and overlapped with those in the Class II group. It is known that Class II and Class III malocclusions have a complex aetiology, related – among the other environmental factors – to different genetic factors that are still largely unexplored^35,36^. Therefore, the present results could suggest that only the craniofacial morphologic characteristics of Class II malocclusions are associated with alterations in the shape of the sella turcica. A possible explanation for our results, in light of these considerations, could be that the genetic alterations – with different expressions – of the development of the maxillary or mandibular bone can also have an expression at the level of the sella turcica. Further studies are needed for an in-depth analysis of such aspects, and to clinically prove if the observation of a posteriorly displaced posterior clinoidal process, a flattened floor of the sella and a concave anterior wall (Fig. 6A) could predict the possible development of a skeletal Class II malocclusion in a young subject.

As this is the first study to report a geometric morphometric analysis of the shape of the sella in growing patients with different skeletal sagittal classes, no comparison with the existing literature can be made. In a study of 151 subjects aged 8–16 years, Kucia *et al*.^11^ reported a more distal position of jaw structures in patients with sella turcica anomalies, consisting mainly in sella turcica bridging; however, these results are not comparable to those of the present study as Kucia *et al*. analysed the sella shape through a qualitative subjective assessment. Alkofide *et al*.^1^ evaluated the sella shape using the morphologic classification by Axelsson *et al*.^31^ in Class I, Class II, and Class III subjects, but they only reported that 67% of the studied subjects had a normal sella shape. Therefore, a description of the sella turcica shape among different malocclusions like the one reported in the present study, has never been presented before.

Regarding the linear measurements of the sella turcica size, an autoptic anatomical study of 250 sphenoidal bones of cadavers of different ages by Quakinine and Hardy^37^ found that the average sella length was 8 mm and the average depth was 6 mm. These reported values for the sella length are in accordance with the results of the present study; however, we found a greater sella depth compared with the values reported by Quakinine and Hardy (Table 4)^37^. Regarding the influence of gender, Alkofide *et al*.^1^ found no significant effect, while Silverman^38^ reported larger sella dimensions in male subjects compared with females. In this present study, the three groups showed a comparable gender distribution and no differences were found when comparing the linear measurements pooled by gender. Regarding the effect of age, it is accepted that the growth rate of the sella turcica decreases rapidly after the first few years of life, and increases again during puberty, then slows down and ceases during early adulthood^19^. In fact, several authors have observed larger sella dimensions in older patients^1,39^, and the sella cephalometric landmark (the centre of the sella) is displaced downward and backward during growth due to the enlargement of the sella, which is mainly caused by bone resorption at the posterior wall of the sella^40^. In the present study, to avoid the influence of age as a confounding factor, the age of participants was restricted to a narrow range of 9 to 13 years of age. The decision to select a sample of growing subjects instead of adults was based on the fact that the orthopaedic treatment of skeletal malocclusion is initiated in the pre-pubertal stage; therefore, information acquired from subjects in the same age range of treated patients would be more meaningful from a clinical point of view. A statistically significant difference regarding the linear measurements of the sella depth and sella length was observed when comparing subjects of 9–10 years of age with patients of 13 years of age, confirming what is reported in the literature regarding the enlargement of the sella during growth. These results did not alter the comparison of the sella linear measurements between different skeletal classes, since the age distribution between these groups was comparable.

In the present study, no difference was found in terms of the sella length and depth between subjects classified as Class I, Class II, and Class III. This is in accordance with the findings of Preston^39^ and those of Alkofide *et al*.^1^. Moreover, Alkofide *et al*.^1^ measured an additional dimension, the sella diameter (defined as the distance between the tuberculum sellae and the most posterior point of the posterior wall of the sella), which differed significantly between subjects with different skeletal sagittal classes, with larger diameters observed in skeletal Class III subjects and smaller diameters in skeletal Class II subjects.

Regarding the limitations of the present study, due to the retrospective nature, care was taken during the selection process by blinding the operator to the aim of the study and using a rigid chronological order. The main limitation could be the use of bi-dimensional images, since modern three-dimensional imaging techniques provide more information. However, plain lateral cephalograms are still the most commonly used diagnostic technique for orthodontic patients, therefore our results could easily be transferred to the everyday orthodontic practice.

Regarding the clinical significance of the present study, it is known that some craniofacial anomalies and developmental alterations are associated with changes in the shape of the sella. Skeletal malocclusions are caused by alterations in the growth of the maxilla, the mandible, or both, and they share a common embryological origin with the anterior part of the sella turcica. Only the dimensions of the sella change with age^1,39^, as the shape is not altered during growth^6^ (established during the first years of life), and therefore the sella shape could potentially be used as a predictor of craniofacial growth. In the present study, we found differences between subjects with different sagittal patterns of craniofacial growth using a precise quantitative method, representing the first report of this kind in the literature. Further studies are needed to deepen our understanding of this topic, as well as to evaluate how a geometric morphometric assessment of the shape of the sella turcica could be used as a predictor of craniofacial growth.

## Conclusions

A statistically significant difference in the sella shape was found between subjects with skeletal Class I and subjects with skeletal Class II in a population aged 9–13 years, as evaluated by a geometric morphometric analysis. No differences in sella length or depth were found between the three groups, while an enlargement of the sella – not altering its shape – was found between subjects of 13 years of age compared with subjects of 9–10 years of age.

## Supplementary information

Dataset 1

## Data Availability

All data generated or analysed during this study are included in this published article (and the supplementary information files). Any other material is available from the corresponding author upon reasonable request.
