# Supplementary material for: Morphometric analysis of sella turcica in growing patients: an observational study on shape and dimensions in different sagittal craniofacial patterns
Source: Sci Rep. 2019 Dec 17;9:19309. doi: 10.1038/s41598-019-55916-y (PMC6917804; doi:10.1038/s41598-019-55916-y)
Supplement: Supplementary file 1 — Dataset 1 [file 41598_2019_55916_MOESM1_ESM.docx]

**Original article**

**Morphometric analysis of sella turcica in growing patients: an observational study on shape and dimensions in different sagittal craniofacial patterns.**

***Running title: Morphometric analysis of sella turcica***

Michele **Tepedino**^1^*, Michele **Laurenziello**^2^, Laura **Guida**^2^, Graziano **Montaruli**^2^, Giuseppe **Troiano**^2^, Claudio **Chimenti**^1^, Marco **Colonna**^2^ & Domenico **Ciavarella**^2^

^1^ Department of Biotechnological and Applied Clinical Sciences, University of L’Aquila, L’Aquila, Italy.

^2^ Department of Clinical and Experimental Medicine, University of Foggia, Foggia, Italy.

***Correspondence to**: Dr Michele Tepedino
 Department of Biotechnological and Applied Clinical Sciences
 University of L’Aquila
 V.le S. Salvatore, Edificio Delta 6
 67100 L’Aquila (AQ), Italy
 E-mail: [m.tepedino@hotmail.it](mailto:m.tepedino@hotmail.it)
 Phone: +39 0862 434782

LM=6

2115.00000 1968.00000

2139.00000 1960.00000

2121.00000 1920.00000

2160.00000 1885.00000

2229.00000 1965.00000

2200.00000 2026.00000

CURVES=3

POINTS=20

2142.00000 1966.00000

2138.00000 1953.00000

2130.00000 1942.00000

2124.00000 1930.00000

2120.00000 1917.00000

2123.00000 1904.00000

2132.00000 1894.00000

2144.00000 1890.00000

2158.00000 1887.00000

2172.00000 1886.00000

2184.00000 1891.00000

2196.00000 1897.00000

2209.00000 1901.00000

2218.00000 1912.00000

2226.00000 1922.00000

2230.00000 1935.00000

2232.00000 1949.00000

2231.00000 1963.00000

2227.00000 1976.00000

2226.00000 1990.00000

POINTS=5

2146.00000 1970.00000

2141.00000 1980.00000

2129.00000 1984.00000

2118.00000 1979.00000

2114.00000 1970.00000

POINTS=5

2226.00000 1991.00000

2226.00000 2003.00000

2215.00000 2009.00000

2205.00000 2015.00000

2196.00000 2024.00000

IMAGE=C:\Users\Pc\Desktop\tutte rx in ordine\01.JPG

ID=0

SCALE=0.080470

LM=6

1329.00000 997.00000

1354.00000 994.00000

1352.00000 966.00000

1398.00000 947.00000

1435.00000 991.00000

1418.00000 1006.00000

CURVES=3

POINTS=20

1351.00000 993.00000

1350.00000 985.00000

1347.00000 977.00000

1346.00000 969.00000

1349.00000 961.00000

1355.00000 955.00000

1363.00000 953.00000

1371.00000 950.00000

1379.00000 947.00000

1387.00000 945.00000

1396.00000 945.00000

1404.00000 947.00000

1412.00000 949.00000

1419.00000 954.00000

1426.00000 959.00000

1428.00000 961.00000

1432.00000 968.00000

1435.00000 975.00000

1439.00000 983.00000

1439.00000 991.00000

POINTS=5

1355.00000 996.00000

1350.00000 1000.00000

1346.00000 1005.00000

1340.00000 1006.00000

1335.00000 1002.00000

POINTS=5

1434.00000 995.00000

1427.00000 994.00000

1422.00000 997.00000

1417.00000 1001.00000

1414.00000 1007.00000

IMAGE=C:\Users\Pc\Desktop\tutte rx in ordine\02.JPG

ID=1

SCALE=0.123929

LM=6

341.00000 468.00000

350.00000 466.00000

340.00000 451.00000

355.00000 435.00000

374.00000 451.00000

366.00000 464.00000

CURVES=3

POINTS=20

349.00000 467.00000

347.00000 464.00000

344.00000 460.00000

342.00000 458.00000

340.00000 454.00000

339.00000 450.00000

339.00000 446.00000

340.00000 442.00000

342.00000 438.00000

346.00000 436.00000

349.00000 434.00000

353.00000 435.00000

357.00000 434.00000

362.00000 434.00000

365.00000 436.00000

369.00000 437.00000

372.00000 440.00000

373.00000 444.00000

373.00000 448.00000

374.00000 452.00000

POINTS=5

349.00000 467.00000

349.00000 472.00000

346.00000 475.00000

343.00000 473.00000

340.00000 469.00000

POINTS=5

375.00000 453.00000

371.00000 455.00000

367.00000 456.00000

365.00000 459.00000

365.00000 463.00000

IMAGE=C:\Users\Pc\Desktop\tutte rx in ordine\03.jpg

ID=2

SCALE=0.263107

LM=6

889.00000 1523.00000

926.00000 1518.00000

914.00000 1474.00000

948.00000 1434.00000

995.00000 1494.00000

982.00000 1515.00000

CURVES=3

POINTS=20

926.00000 1514.00000

920.00000 1505.00000

916.00000 1496.00000

913.00000 1486.00000

912.00000 1476.00000

912.00000 1465.00000

914.00000 1455.00000

920.00000 1446.00000

926.00000 1438.00000

936.00000 1435.00000

946.00000 1431.00000

956.00000 1431.00000

966.00000 1433.00000

975.00000 1438.00000

985.00000 1443.00000

991.00000 1452.00000

994.00000 1461.00000

996.00000 1471.00000

997.00000 1482.00000

997.00000 1492.00000

POINTS=5

926.00000 1520.00000

922.00000 1533.00000

909.00000 1537.00000

896.00000 1534.00000

889.00000 1523.00000

POINTS=5

995.00000 1496.00000

991.00000 1500.00000

986.00000 1503.00000

982.00000 1508.00000

980.00000 1514.00000

IMAGE=C:\Users\Pc\Desktop\tutte rx in ordine\04.jpg

ID=3

SCALE=0.087103

LM=6

1116.00000 2225.00000

1162.00000 2227.00000

1171.00000 2163.00000

1246.00000 2129.00000

1293.00000 2196.00000

1217.00000 2223.00000

CURVES=3

POINTS=20

1163.00000 2223.00000

1163.00000 2210.00000

1164.00000 2196.00000

1167.00000 2183.00000

1170.00000 2170.00000

1176.00000 2159.00000

1184.00000 2149.00000

1194.00000 2140.00000

1205.00000 2133.00000

1216.00000 2128.00000

1229.00000 2124.00000

1243.00000 2123.00000

1255.00000 2128.00000

1267.00000 2134.00000

1276.00000 2143.00000

1283.00000 2154.00000

1289.00000 2166.00000

1287.00000 2179.00000

1289.00000 2192.00000

1285.00000 2205.00000

POINTS=5

1163.00000 2227.00000

1157.00000 2242.00000

1141.00000 2244.00000

1126.00000 2237.00000

1119.00000 2223.00000

POINTS=5

1284.00000 2204.00000

1268.00000 2214.00000

1250.00000 2212.00000

1231.00000 2209.00000

1220.00000 2224.00000

IMAGE=C:\Users\Pc\Desktop\tutte rx in ordine\05.JPG

ID=4

SCALE=0.064828

LM=6

797.00000 563.00000

803.00000 562.00000

800.00000 547.00000

818.00000 538.00000

828.00000 554.00000

821.00000 568.00000

CURVES=3

POINTS=20

803.00000 565.00000

802.00000 562.00000

800.00000 558.00000

800.00000 554.00000

800.00000 550.00000

801.00000 546.00000

803.00000 543.00000

806.00000 540.00000

809.00000 539.00000

812.00000 538.00000

816.00000 537.00000

820.00000 538.00000

824.00000 538.00000

826.00000 540.00000

829.00000 542.00000

830.00000 545.00000

830.00000 549.00000

830.00000 553.00000

828.00000 556.00000

827.00000 560.00000

POINTS=5

803.00000 567.00000

801.00000 569.00000

798.00000 569.00000

797.00000 567.00000

796.00000 564.00000

POINTS=5

827.00000 561.00000

824.00000 562.00000

821.00000 564.00000

818.00000 566.00000

817.00000 569.00000

IMAGE=C:\Users\Pc\Desktop\tutte rx in ordine\06.jpg

ID=5

SCALE=0.333322

LM=6

1629.00000 1211.00000

1658.00000 1205.00000

1645.00000 1174.00000

1686.00000 1141.00000

1725.00000 1179.00000

1676.00000 1214.00000

CURVES=3

POINTS=20

1657.00000 1204.00000

1652.00000 1197.00000

1649.00000 1189.00000

1646.00000 1181.00000

1646.00000 1172.00000

1650.00000 1165.00000

1654.00000 1158.00000

1660.00000 1153.00000

1665.00000 1147.00000

1671.00000 1142.00000

1678.00000 1137.00000

1687.00000 1135.00000

1695.00000 1138.00000

1701.00000 1143.00000

1708.00000 1149.00000

1713.00000 1156.00000

1718.00000 1163.00000

1723.00000 1169.00000

1726.00000 1177.00000

1724.00000 1185.00000

POINTS=5

1660.00000 1206.00000

1659.00000 1216.00000

1649.00000 1218.00000

1640.00000 1213.00000

1634.00000 1206.00000

POINTS=5

1724.00000 1186.00000

1709.00000 1194.00000

1693.00000 1201.00000

1677.00000 1208.00000

1670.00000 1222.00000

IMAGE=C:\Users\Pc\Desktop\tutte rx in ordine\07.JPG

ID=6

SCALE=0.110204

LM=6

1761.00000 1363.00000

1784.00000 1358.00000

1779.00000 1315.00000

1828.00000 1306.00000

1875.00000 1357.00000

1854.00000 1387.00000

CURVES=3

POINTS=20

1785.00000 1364.00000

1782.00000 1354.00000

1779.00000 1344.00000

1776.00000 1334.00000

1776.00000 1323.00000

1781.00000 1314.00000

1788.00000 1306.00000

1799.00000 1304.00000

1809.00000 1301.00000

1819.00000 1301.00000

1830.00000 1305.00000

1840.00000 1306.00000

1849.00000 1311.00000

1858.00000 1317.00000

1866.00000 1324.00000

1871.00000 1334.00000

1873.00000 1344.00000

1876.00000 1354.00000

1878.00000 1364.00000

1877.00000 1374.00000

POINTS=5

1786.00000 1370.00000

1781.00000 1378.00000

1771.00000 1378.00000

1762.00000 1373.00000

1758.00000 1364.00000

POINTS=5

1873.00000 1376.00000

1869.00000 1376.00000

1862.00000 1376.00000

1857.00000 1381.00000

1855.00000 1388.00000

IMAGE=C:\Users\Pc\Desktop\tutte rx in ordine\08.JPG

ID=7

SCALE=0.096153

LM=6

1571.00000 1144.00000

1597.00000 1142.00000

1597.00000 1090.00000

1650.00000 1063.00000

1701.00000 1101.00000

1674.00000 1124.00000

CURVES=3

POINTS=20

1594.00000 1138.00000

1591.00000 1128.00000

1588.00000 1117.00000

1587.00000 1107.00000

1589.00000 1096.00000

1595.00000 1087.00000

1600.00000 1077.00000

1608.00000 1071.00000

1617.00000 1066.00000

1627.00000 1062.00000

1637.00000 1060.00000

1648.00000 1060.00000

1659.00000 1060.00000

1670.00000 1060.00000

1678.00000 1066.00000

1686.00000 1074.00000

1692.00000 1080.00000

1696.00000 1090.00000

1702.00000 1099.00000

1704.00000 1109.00000

POINTS=5

1597.00000 1143.00000

1599.00000 1153.00000

1594.00000 1160.00000

1585.00000 1156.00000

1578.00000 1149.00000

POINTS=5

1703.00000 1108.00000

1693.00000 1112.00000

1681.00000 1112.00000

1673.00000 1119.00000

1674.00000 1130.00000

IMAGE=C:\Users\Pc\Desktop\tutte rx in ordine\09.JPG

ID=8

SCALE=0.100249

LM=6

700.00000 593.00000

714.00000 591.00000

710.00000 579.00000

728.00000 567.00000

744.00000 587.00000

731.00000 599.00000

CURVES=3

POINTS=20

714.00000 593.00000

713.00000 589.00000

711.00000 585.00000

710.00000 581.00000

710.00000 577.00000

712.00000 574.00000

714.00000 571.00000

717.00000 568.00000

721.00000 568.00000

724.00000 565.00000

728.00000 565.00000

731.00000 567.00000

734.00000 569.00000

738.00000 571.00000

741.00000 574.00000

743.00000 576.00000

744.00000 580.00000

745.00000 584.00000

746.00000 588.00000

745.00000 592.00000

POINTS=5

713.00000 593.00000

712.00000 598.00000

707.00000 600.00000

703.00000 598.00000

699.00000 595.00000

POINTS=5

745.00000 592.00000

740.00000 592.00000

736.00000 594.00000

731.00000 596.00000

729.00000 600.00000

IMAGE=C:\Users\Pc\Desktop\tutte rx in ordine\10.jpg

ID=9

SCALE=0.237975

LM=6

1732.00000 1627.00000

1784.00000 1625.00000

1767.00000 1574.00000

1831.00000 1551.00000

1880.00000 1589.00000

1833.00000 1626.00000

CURVES=3

POINTS=20

1785.00000 1619.00000

1780.00000 1609.00000

1775.00000 1600.00000

1767.00000 1592.00000

1765.00000 1581.00000

1770.00000 1571.00000

1778.00000 1563.00000

1785.00000 1555.00000

1796.00000 1552.00000

1806.00000 1548.00000

1817.00000 1546.00000

1828.00000 1545.00000

1839.00000 1547.00000

1850.00000 1549.00000

1861.00000 1553.00000

1870.00000 1560.00000

1875.00000 1569.00000

1880.00000 1579.00000

1885.00000 1589.00000

1883.00000 1599.00000

POINTS=5

1788.00000 1624.00000

1780.00000 1640.00000

1766.00000 1650.00000

1748.00000 1643.00000

1736.00000 1629.00000

POINTS=5

1885.00000 1610.00000

1871.00000 1606.00000

1856.00000 1606.00000

1845.00000 1615.00000

1835.00000 1624.00000

IMAGE=C:\Users\Pc\Desktop\tutte rx in ordine\11.JPG

ID=10

SCALE=0.083400

LM=6

1001.00000 1555.00000

1049.00000 1548.00000

1024.00000 1500.00000

1068.00000 1467.00000

1123.00000 1514.00000

1107.00000 1542.00000

CURVES=3

POINTS=20

1056.00000 1548.00000

1050.00000 1539.00000

1044.00000 1529.00000

1040.00000 1518.00000

1032.00000 1510.00000

1027.00000 1500.00000

1025.00000 1488.00000

1026.00000 1477.00000

1033.00000 1468.00000

1045.00000 1465.00000

1056.00000 1465.00000

1068.00000 1466.00000

1080.00000 1465.00000

1091.00000 1469.00000

1101.00000 1474.00000

1110.00000 1482.00000

1117.00000 1491.00000

1120.00000 1502.00000

1124.00000 1513.00000

1123.00000 1523.00000

POINTS=5

1060.00000 1552.00000

1052.00000 1567.00000

1036.00000 1575.00000

1019.00000 1569.00000

1005.00000 1557.00000

POINTS=5

1123.00000 1523.00000

1119.00000 1529.00000

1112.00000 1532.00000

1105.00000 1534.00000

1099.00000 1537.00000

IMAGE=C:\Users\Pc\Desktop\tutte rx in ordine\12.jpg

ID=11

SCALE=0.091902

LM=6

1653.00000 1364.00000

1680.00000 1364.00000

1671.00000 1318.00000

1713.00000 1297.00000

1748.00000 1333.00000

1733.00000 1346.00000

CURVES=3

POINTS=20

1677.00000 1361.00000

1675.00000 1353.00000

1671.00000 1346.00000

1669.00000 1338.00000

1668.00000 1330.00000

1670.00000 1322.00000

1673.00000 1314.00000

1678.00000 1308.00000

1686.00000 1303.00000

1693.00000 1300.00000

1700.00000 1296.00000

1708.00000 1294.00000

1717.00000 1294.00000

1725.00000 1296.00000

1733.00000 1298.00000

1741.00000 1301.00000

1747.00000 1306.00000

1750.00000 1314.00000

1752.00000 1322.00000

1750.00000 1330.00000

POINTS=5

1678.00000 1364.00000

1677.00000 1374.00000

1669.00000 1377.00000

1660.00000 1372.00000

1655.00000 1364.00000

POINTS=5

1751.00000 1332.00000

1744.00000 1332.00000

1738.00000 1336.00000

1733.00000 1341.00000

1732.00000 1347.00000

IMAGE=C:\Users\Pc\Desktop\tutte rx in ordine\13.JPG

ID=12

SCALE=0.101502

LM=6

1692.00000 1553.00000

1722.00000 1546.00000

1698.00000 1485.00000

1766.00000 1458.00000

1829.00000 1517.00000

1804.00000 1536.00000

CURVES=3

POINTS=20

1718.00000 1547.00000

1712.00000 1534.00000

1704.00000 1523.00000

1698.00000 1510.00000

1697.00000 1497.00000

1699.00000 1483.00000

1707.00000 1473.00000

1719.00000 1465.00000

1731.00000 1459.00000

1744.00000 1455.00000

1757.00000 1455.00000

1771.00000 1457.00000

1784.00000 1461.00000

1797.00000 1466.00000

1808.00000 1470.00000

1817.00000 1480.00000

1824.00000 1491.00000

1830.00000 1503.00000

1834.00000 1516.00000

1834.00000 1530.00000

POINTS=5

1721.00000 1549.00000

1719.00000 1560.00000

1710.00000 1568.00000

1699.00000 1565.00000

1693.00000 1556.00000

POINTS=5

1833.00000 1532.00000

1825.00000 1531.00000

1817.00000 1530.00000

1809.00000 1532.00000

1804.00000 1538.00000

IMAGE=C:\Users\Pc\Desktop\tutte rx in ordine\14.JPG

ID=13

SCALE=0.085458

LM=6

1041.00000 1491.00000

1077.00000 1483.00000

1073.00000 1438.00000

1116.00000 1406.00000

1178.00000 1466.00000

1146.00000 1504.00000

CURVES=3

POINTS=20

1084.00000 1491.00000

1077.00000 1481.00000

1071.00000 1470.00000

1069.00000 1457.00000

1067.00000 1445.00000

1068.00000 1432.00000

1076.00000 1424.00000

1087.00000 1418.00000

1097.00000 1411.00000

1109.00000 1408.00000

1121.00000 1405.00000

1134.00000 1406.00000

1146.00000 1409.00000

1157.00000 1414.00000

1165.00000 1424.00000

1173.00000 1433.00000

1176.00000 1446.00000

1178.00000 1458.00000

1177.00000 1471.00000

1174.00000 1483.00000

POINTS=5

1088.00000 1489.00000

1085.00000 1506.00000

1068.00000 1512.00000

1051.00000 1506.00000

1043.00000 1489.00000

POINTS=5

1174.00000 1483.00000

1163.00000 1484.00000

1152.00000 1487.00000

1144.00000 1495.00000

1142.00000 1506.00000

IMAGE=C:\Users\Pc\Desktop\tutte rx in ordine\15.jpg

ID=14

SCALE=0.089559

LM=6

967.00000 1537.00000

987.00000 1536.00000

988.00000 1499.00000

1024.00000 1473.00000

1079.00000 1515.00000

1073.00000 1550.00000

CURVES=3

POINTS=20

989.00000 1542.00000

985.00000 1532.00000

982.00000 1522.00000

982.00000 1511.00000

986.00000 1501.00000

991.00000 1491.00000

996.00000 1482.00000

1005.00000 1478.00000

1015.00000 1474.00000

1025.00000 1472.00000

1036.00000 1472.00000

1047.00000 1473.00000

1057.00000 1477.00000

1066.00000 1482.00000

1072.00000 1491.00000

1078.00000 1500.00000

1081.00000 1510.00000

1087.00000 1519.00000

1090.00000 1529.00000

1092.00000 1539.00000

POINTS=5

992.00000 1545.00000

989.00000 1553.00000

982.00000 1553.00000

975.00000 1549.00000

971.00000 1541.00000

POINTS=5

1094.00000 1536.00000

1086.00000 1540.00000

1076.00000 1542.00000

1068.00000 1546.00000

1062.00000 1553.00000

IMAGE=C:\Users\Pc\Desktop\tutte rx in ordine\16.jpg

ID=15

SCALE=0.089578

LM=6

1724.00000 1177.00000

1758.00000 1177.00000

1717.00000 1138.00000

1775.00000 1102.00000

1823.00000 1148.00000

1792.00000 1174.00000

CURVES=3

POINTS=20

1753.00000 1176.00000

1743.00000 1170.00000

1734.00000 1163.00000

1727.00000 1155.00000

1718.00000 1146.00000

1719.00000 1135.00000

1724.00000 1124.00000

1732.00000 1116.00000

1742.00000 1109.00000

1753.00000 1105.00000

1765.00000 1101.00000

1776.00000 1100.00000

1788.00000 1098.00000

1800.00000 1102.00000

1808.00000 1110.00000

1810.00000 1122.00000

1814.00000 1133.00000

1817.00000 1144.00000

1812.00000 1155.00000

1804.00000 1164.00000

POINTS=5

1754.00000 1179.00000

1752.00000 1190.00000

1742.00000 1191.00000

1732.00000 1186.00000

1724.00000 1179.00000

POINTS=5

1802.00000 1164.00000

1795.00000 1166.00000

1788.00000 1169.00000

1782.00000 1173.00000

1781.00000 1181.00000

IMAGE=C:\Users\Pc\Desktop\tutte rx in ordine\17.JPG

ID=16

SCALE=0.104706

LM=6

1835.00000 1511.00000

1878.00000 1495.00000

1826.00000 1440.00000

1888.00000 1403.00000

1940.00000 1454.00000

1910.00000 1490.00000

CURVES=3

POINTS=20

1873.00000 1489.00000

1863.00000 1481.00000

1857.00000 1470.00000

1849.00000 1460.00000

1839.00000 1451.00000

1831.00000 1441.00000

1833.00000 1428.00000

1843.00000 1420.00000

1854.00000 1413.00000

1866.00000 1411.00000

1879.00000 1409.00000

1892.00000 1409.00000

1905.00000 1410.00000

1918.00000 1413.00000

1930.00000 1415.00000

1941.00000 1422.00000

1946.00000 1434.00000

1947.00000 1447.00000

1948.00000 1460.00000

1947.00000 1471.00000

POINTS=5

1879.00000 1496.00000

1876.00000 1509.00000

1865.00000 1514.00000

1853.00000 1512.00000

1841.00000 1509.00000

POINTS=5

1948.00000 1467.00000

1938.00000 1470.00000

1930.00000 1478.00000

1920.00000 1481.00000

1912.00000 1490.00000

IMAGE=C:\Users\Pc\Desktop\tutte rx in ordine\18.JPG

ID=17

SCALE=0.084033

LM=6

1407.00000 1586.00000

1444.00000 1571.00000

1444.00000 1526.00000

1491.00000 1494.00000

1543.00000 1546.00000

1495.00000 1582.00000

CURVES=3

POINTS=20

1446.00000 1567.00000

1439.00000 1558.00000

1435.00000 1547.00000

1432.00000 1536.00000

1433.00000 1525.00000

1439.00000 1515.00000

1447.00000 1508.00000

1457.00000 1503.00000

1466.00000 1497.00000

1478.00000 1497.00000

1489.00000 1494.00000

1500.00000 1495.00000

1511.00000 1500.00000

1522.00000 1504.00000

1528.00000 1513.00000

1533.00000 1523.00000

1539.00000 1533.00000

1540.00000 1544.00000

1538.00000 1555.00000

1531.00000 1561.00000

POINTS=5

1448.00000 1576.00000

1442.00000 1592.00000

1431.00000 1604.00000

1415.00000 1602.00000

1407.00000 1588.00000

POINTS=5

1528.00000 1560.00000

1517.00000 1561.00000

1506.00000 1566.00000

1498.00000 1573.00000

1493.00000 1583.00000

IMAGE=C:\Users\Pc\Desktop\tutte rx in ordine\19.JPG

ID=18

SCALE=0.086499

LM=6

1136.00000 1612.00000

1183.00000 1605.00000

1172.00000 1543.00000

1247.00000 1505.00000

1297.00000 1543.00000

1250.00000 1580.00000

CURVES=3

POINTS=20

1180.00000 1603.00000

1172.00000 1593.00000

1169.00000 1581.00000

1169.00000 1568.00000

1168.00000 1556.00000

1168.00000 1543.00000

1175.00000 1533.00000

1184.00000 1524.00000

1194.00000 1518.00000

1205.00000 1512.00000

1218.00000 1510.00000

1229.00000 1504.00000

1241.00000 1502.00000

1254.00000 1502.00000

1266.00000 1505.00000

1277.00000 1510.00000

1288.00000 1518.00000

1294.00000 1528.00000

1297.00000 1540.00000

1300.00000 1552.00000

POINTS=5

1184.00000 1606.00000

1177.00000 1623.00000

1163.00000 1630.00000

1147.00000 1621.00000

1136.00000 1605.00000

POINTS=5

1297.00000 1549.00000

1283.00000 1556.00000

1267.00000 1558.00000

1254.00000 1566.00000

1249.00000 1581.00000

IMAGE=C:\Users\Pc\Desktop\tutte rx in ordine\20.jpg

ID=19

SCALE=0.089477

LM=6

1427.00000 1367.00000

1474.00000 1356.00000

1432.00000 1304.00000

1533.00000 1258.00000

1596.00000 1314.00000

1566.00000 1392.00000

CURVES=3

POINTS=20

1462.00000 1358.00000

1456.00000 1343.00000

1445.00000 1330.00000

1438.00000 1316.00000

1440.00000 1300.00000

1452.00000 1289.00000

1466.00000 1281.00000

1480.00000 1273.00000

1495.00000 1266.00000

1510.00000 1260.00000

1527.00000 1258.00000

1543.00000 1260.00000

1557.00000 1267.00000

1571.00000 1276.00000

1584.00000 1286.00000

1593.00000 1301.00000

1594.00000 1317.00000

1597.00000 1333.00000

1598.00000 1349.00000

1594.00000 1365.00000

POINTS=5

1467.00000 1365.00000

1462.00000 1380.00000

1447.00000 1386.00000

1432.00000 1380.00000

1424.00000 1367.00000

POINTS=5

1590.00000 1371.00000

1579.00000 1375.00000

1568.00000 1382.00000

1557.00000 1389.00000

1555.00000 1400.00000

IMAGE=C:\Users\Pc\Desktop\tutte rx in ordine\21.JPG

ID=20

SCALE=0.096851

LM=6

1763.00000 1718.00000

1810.00000 1711.00000

1774.00000 1680.00000

1830.00000 1627.00000

1879.00000 1671.00000

1841.00000 1683.00000

CURVES=3

POINTS=20

1810.00000 1708.00000

1799.00000 1703.00000

1789.00000 1697.00000

1780.00000 1691.00000

1775.00000 1680.00000

1774.00000 1669.00000

1779.00000 1659.00000

1784.00000 1649.00000

1790.00000 1640.00000

1799.00000 1633.00000

1810.00000 1629.00000

1822.00000 1625.00000

1833.00000 1624.00000

1845.00000 1626.00000

1855.00000 1631.00000

1866.00000 1636.00000

1873.00000 1645.00000

1878.00000 1655.00000

1882.00000 1666.00000

1879.00000 1677.00000

POINTS=5

1809.00000 1712.00000

1804.00000 1727.00000

1791.00000 1731.00000

1776.00000 1729.00000

1764.00000 1720.00000

POINTS=5

1878.00000 1676.00000

1868.00000 1673.00000

1857.00000 1674.00000

1847.00000 1678.00000

1838.00000 1685.00000

IMAGE=C:\Users\Pc\Desktop\tutte rx in ordine\22.JPG

ID=21

SCALE=0.112044

LM=6

33.00000 184.00000

38.00000 184.00000

36.00000 178.00000

44.00000 174.00000

52.00000 182.00000

50.00000 188.00000

CURVES=3

POINTS=20

39.00000 187.00000

38.00000 186.00000

37.00000 184.00000

37.00000 183.00000

37.00000 181.00000

37.00000 179.00000

38.00000 178.00000

39.00000 177.00000

41.00000 176.00000

42.00000 176.00000

44.00000 176.00000

46.00000 177.00000

47.00000 177.00000

49.00000 177.00000

50.00000 178.00000

51.00000 180.00000

51.00000 181.00000

52.00000 183.00000

52.00000 185.00000

51.00000 186.00000

POINTS=5

39.00000 188.00000

38.00000 189.00000

36.00000 189.00000

34.00000 188.00000

33.00000 187.00000

POINTS=5

50.00000 186.00000

49.00000 186.00000

48.00000 186.00000

47.00000 187.00000

46.00000 188.00000

IMAGE=C:\Users\Pc\Desktop\tutte rx in ordine\23.PNG

ID=22

SCALE=0.588172

LM=6

398.00000 496.00000

405.00000 493.00000

399.00000 479.00000

412.00000 471.00000

429.00000 490.00000

423.00000 501.00000

CURVES=3

POINTS=20

406.00000 492.00000

403.00000 489.00000

401.00000 487.00000

399.00000 483.00000

398.00000 480.00000

398.00000 476.00000

399.00000 473.00000

403.00000 471.00000

406.00000 470.00000

410.00000 470.00000

414.00000 470.00000

417.00000 469.00000

421.00000 471.00000

424.00000 474.00000

425.00000 477.00000

426.00000 480.00000

429.00000 484.00000

430.00000 487.00000

432.00000 490.00000

432.00000 494.00000

POINTS=5

408.00000 495.00000

406.00000 499.00000

403.00000 500.00000

399.00000 499.00000

397.00000 496.00000

POINTS=5

431.00000 495.00000

426.00000 495.00000

422.00000 495.00000

419.00000 498.00000

419.00000 503.00000

IMAGE=C:\Users\Pc\Desktop\tutte rx in ordine\24.jpg

ID=23

SCALE=0.263152

LM=6

123.00000 260.00000

130.00000 259.00000

127.00000 249.00000

136.00000 243.00000

146.00000 252.00000

141.00000 258.00000

CURVES=3

POINTS=20

130.00000 259.00000

129.00000 257.00000

128.00000 254.00000

127.00000 252.00000

127.00000 250.00000

126.00000 248.00000

127.00000 246.00000

127.00000 244.00000

129.00000 242.00000

132.00000 243.00000

134.00000 243.00000

136.00000 244.00000

138.00000 243.00000

140.00000 244.00000

142.00000 246.00000

144.00000 246.00000

144.00000 249.00000

146.00000 251.00000

146.00000 253.00000

145.00000 255.00000

POINTS=5

130.00000 260.00000

129.00000 262.00000

126.00000 264.00000

124.00000 262.00000

122.00000 260.00000

POINTS=5

144.00000 255.00000

142.00000 255.00000

140.00000 257.00000

138.00000 258.00000

139.00000 260.00000

IMAGE=C:\Users\Pc\Desktop\tutte rx in ordine\25.PNG

ID=24

SCALE=0.457540

LM=6

286.00000 462.00000

297.00000 463.00000

300.00000 444.00000

312.00000 439.00000

330.00000 457.00000

318.00000 472.00000

CURVES=3

POINTS=20

297.00000 460.00000

297.00000 456.00000

297.00000 453.00000

298.00000 449.00000

298.00000 446.00000

300.00000 442.00000

303.00000 441.00000

306.00000 440.00000

309.00000 440.00000

313.00000 440.00000

317.00000 440.00000

320.00000 440.00000

322.00000 443.00000

325.00000 444.00000

327.00000 448.00000

329.00000 451.00000

329.00000 454.00000

330.00000 458.00000

330.00000 461.00000

331.00000 465.00000

POINTS=5

297.00000 462.00000

295.00000 466.00000

292.00000 469.00000

288.00000 467.00000

286.00000 464.00000

POINTS=5

332.00000 468.00000

328.00000 471.00000

321.00000 471.00000

314.00000 473.00000

311.00000 478.00000

IMAGE=C:\Users\Pc\Desktop\tutte rx in ordine\26.jpg

ID=25

SCALE=0.259735

LM=6

252.00000 396.00000

263.00000 396.00000

263.00000 382.00000

279.00000 373.00000

296.00000 392.00000

288.00000 406.00000

CURVES=3

POINTS=20

262.00000 395.00000

261.00000 392.00000

259.00000 388.00000

258.00000 384.00000

258.00000 380.00000

261.00000 378.00000

263.00000 374.00000

266.00000 374.00000

270.00000 372.00000

274.00000 371.00000

277.00000 373.00000

281.00000 372.00000

284.00000 372.00000

288.00000 374.00000

291.00000 375.00000

294.00000 378.00000

295.00000 382.00000

295.00000 386.00000

296.00000 390.00000

296.00000 394.00000

POINTS=5

263.00000 397.00000

260.00000 401.00000

256.00000 404.00000

253.00000 401.00000

251.00000 396.00000

POINTS=5

296.00000 394.00000

292.00000 398.00000

287.00000 400.00000

283.00000 404.00000

282.00000 409.00000

IMAGE=C:\Users\Pc\Desktop\tutte rx in ordine\27.PNG

ID=26

SCALE=0.336087

LM=6

341.00000 472.00000

357.00000 472.00000

355.00000 456.00000

366.00000 452.00000

380.00000 470.00000

372.00000 479.00000

CURVES=3

POINTS=20

354.00000 470.00000

354.00000 467.00000

354.00000 464.00000

354.00000 461.00000

355.00000 458.00000

355.00000 456.00000

358.00000 454.00000

360.00000 452.00000

363.00000 452.00000

366.00000 453.00000

369.00000 453.00000

371.00000 454.00000

373.00000 456.00000

375.00000 459.00000

377.00000 460.00000

379.00000 463.00000

380.00000 465.00000

381.00000 468.00000

381.00000 471.00000

380.00000 474.00000

POINTS=5

353.00000 472.00000

351.00000 476.00000

347.00000 477.00000

344.00000 473.00000

342.00000 469.00000

POINTS=5

380.00000 474.00000

377.00000 475.00000

374.00000 475.00000

372.00000 478.00000

370.00000 480.00000

IMAGE=C:\Users\Pc\Desktop\tutte rx in ordine\28.jpg

ID=27

SCALE=0.263107

LM=6

1516.00000 1585.00000

1555.00000 1580.00000

1545.00000 1549.00000

1599.00000 1520.00000

1646.00000 1577.00000

1628.00000 1611.00000

CURVES=3

POINTS=20

1548.00000 1581.00000

1542.00000 1572.00000

1539.00000 1561.00000

1540.00000 1550.00000

1545.00000 1540.00000

1551.00000 1531.00000

1559.00000 1523.00000

1569.00000 1518.00000

1580.00000 1515.00000

1590.00000 1512.00000

1602.00000 1512.00000

1612.00000 1514.00000

1623.00000 1516.00000

1632.00000 1523.00000

1641.00000 1528.00000

1648.00000 1537.00000

1654.00000 1546.00000

1656.00000 1557.00000

1655.00000 1569.00000

1648.00000 1577.00000

POINTS=5

1549.00000 1584.00000

1544.00000 1592.00000

1534.00000 1594.00000

1525.00000 1590.00000

1518.00000 1582.00000

POINTS=5

1643.00000 1577.00000

1633.00000 1583.00000

1624.00000 1590.00000

1617.00000 1600.00000

1622.00000 1609.00000

IMAGE=C:\Users\Pc\Desktop\tutte rx in ordine\29.jpg

ID=28

SCALE=0.087326

LM=6

1468.00000 1648.00000

1496.00000 1641.00000

1450.00000 1569.00000

1526.00000 1509.00000

1593.00000 1574.00000

1549.00000 1611.00000

CURVES=3

POINTS=20

1498.00000 1634.00000

1486.00000 1623.00000

1475.00000 1611.00000

1463.00000 1600.00000

1452.00000 1587.00000

1450.00000 1570.00000

1454.00000 1554.00000

1462.00000 1539.00000

1471.00000 1526.00000

1484.00000 1516.00000

1498.00000 1507.00000

1514.00000 1502.00000

1531.00000 1504.00000

1548.00000 1505.00000

1563.00000 1512.00000

1576.00000 1522.00000

1586.00000 1535.00000

1592.00000 1551.00000

1593.00000 1568.00000

1594.00000 1585.00000

POINTS=5

1501.00000 1634.00000

1501.00000 1649.00000

1493.00000 1660.00000

1479.00000 1657.00000

1471.00000 1644.00000

POINTS=5

1595.00000 1585.00000

1577.00000 1588.00000

1558.00000 1590.00000

1544.00000 1601.00000

1547.00000 1618.00000

IMAGE=C:\Users\Pc\Desktop\tutte rx in ordine\30.jpg

ID=29

SCALE=0.089229

LM=6

1700.00000 991.00000

1715.00000 991.00000

1709.00000 961.00000

1749.00000 935.00000

1785.00000 967.00000

1762.00000 995.00000

CURVES=3

POINTS=20

1715.00000 986.00000

1708.00000 982.00000

1705.00000 975.00000

1705.00000 967.00000

1706.00000 958.00000

1709.00000 951.00000

1715.00000 945.00000

1721.00000 940.00000

1727.00000 936.00000

1734.00000 932.00000

1742.00000 930.00000

1750.00000 930.00000

1757.00000 932.00000

1765.00000 935.00000

1773.00000 938.00000

1777.00000 944.00000

1780.00000 952.00000

1784.00000 959.00000

1785.00000 967.00000

1786.00000 975.00000

POINTS=5

1720.00000 996.00000

1717.00000 1002.00000

1711.00000 1001.00000

1705.00000 997.00000

1703.00000 991.00000

POINTS=5

1786.00000 973.00000

1778.00000 979.00000

1769.00000 982.00000

1762.00000 989.00000

1760.00000 998.00000

IMAGE=C:\Users\Pc\Desktop\tutte rx in ordine\31.JPG

ID=30

SCALE=0.140132

LM=6

1294.00000 2053.00000

1327.00000 2053.00000

1338.00000 2004.00000

1393.00000 1983.00000

1420.00000 2032.00000

1396.00000 2075.00000

CURVES=3

POINTS=20

1324.00000 2046.00000

1325.00000 2036.00000

1326.00000 2026.00000

1330.00000 2016.00000

1332.00000 2006.00000

1340.00000 1999.00000

1348.00000 1994.00000

1355.00000 1987.00000

1365.00000 1983.00000

1375.00000 1981.00000

1384.00000 1977.00000

1394.00000 1979.00000

1403.00000 1983.00000

1410.00000 1990.00000

1413.00000 2000.00000

1418.00000 2008.00000

1421.00000 2018.00000

1423.00000 2028.00000

1424.00000 2038.00000

1422.00000 2048.00000

POINTS=5

1326.00000 2056.00000

1319.00000 2066.00000

1308.00000 2071.00000

1297.00000 2065.00000

1293.00000 2052.00000

POINTS=5

1422.00000 2045.00000

1411.00000 2050.00000

1401.00000 2056.00000

1392.00000 2064.00000

1390.00000 2075.00000

IMAGE=C:\Users\Pc\Desktop\tutte rx in ordine\32.JPG

ID=31

SCALE=0.097771

LM=6

239.00000 314.00000

246.00000 313.00000

244.00000 304.00000

256.00000 300.00000

269.00000 311.00000

260.00000 320.00000

CURVES=3

POINTS=20

245.00000 313.00000

244.00000 310.00000

244.00000 308.00000

243.00000 305.00000

243.00000 303.00000

244.00000 300.00000

246.00000 299.00000

248.00000 298.00000

251.00000 298.00000

253.00000 297.00000

256.00000 297.00000

258.00000 299.00000

260.00000 299.00000

263.00000 301.00000

265.00000 302.00000

266.00000 304.00000

268.00000 306.00000

269.00000 309.00000

270.00000 310.00000

270.00000 313.00000

POINTS=5

245.00000 314.00000

245.00000 317.00000

242.00000 319.00000

240.00000 316.00000

238.00000 312.00000

POINTS=5

269.00000 313.00000

265.00000 315.00000

262.00000 316.00000

258.00000 317.00000

257.00000 321.00000

IMAGE=C:\Users\Pc\Desktop\tutte rx in ordine\33.PNG

ID=32

SCALE=0.356325

LM=6

1480.00000 1339.00000

1500.00000 1336.00000

1488.00000 1304.00000

1546.00000 1291.00000

1576.00000 1329.00000

1547.00000 1344.00000

CURVES=3

POINTS=20

1498.00000 1336.00000

1497.00000 1327.00000

1492.00000 1321.00000

1487.00000 1314.00000

1488.00000 1305.00000

1491.00000 1298.00000

1498.00000 1292.00000

1506.00000 1289.00000

1514.00000 1288.00000

1523.00000 1287.00000

1531.00000 1288.00000

1540.00000 1288.00000

1549.00000 1288.00000

1557.00000 1290.00000

1565.00000 1294.00000

1571.00000 1299.00000

1574.00000 1306.00000

1576.00000 1314.00000

1576.00000 1323.00000

1573.00000 1331.00000

POINTS=5

1497.00000 1337.00000

1495.00000 1343.00000

1489.00000 1346.00000

1483.00000 1345.00000

1480.00000 1339.00000

POINTS=5

1572.00000 1331.00000

1564.00000 1335.00000

1556.00000 1337.00000

1547.00000 1340.00000

1543.00000 1346.00000

IMAGE=C:\Users\Pc\Desktop\tutte rx in ordine\34.JPG

ID=33

SCALE=0.105440

LM=6

851.00000 1510.00000

881.00000 1506.00000

863.00000 1457.00000

905.00000 1428.00000

949.00000 1459.00000

909.00000 1493.00000

CURVES=3

POINTS=20

879.00000 1503.00000

875.00000 1496.00000

871.00000 1488.00000

866.00000 1480.00000

863.00000 1472.00000

862.00000 1463.00000

863.00000 1454.00000

866.00000 1446.00000

871.00000 1438.00000

878.00000 1434.00000

886.00000 1430.00000

894.00000 1426.00000

903.00000 1426.00000

912.00000 1426.00000

921.00000 1428.00000

929.00000 1431.00000

936.00000 1436.00000

941.00000 1443.00000

946.00000 1450.00000

947.00000 1459.00000

POINTS=5

880.00000 1505.00000

877.00000 1516.00000

870.00000 1524.00000

861.00000 1518.00000

854.00000 1509.00000

POINTS=5

945.00000 1461.00000

939.00000 1473.00000

927.00000 1481.00000

914.00000 1484.00000

906.00000 1493.00000

IMAGE=C:\Users\Pc\Desktop\tutte rx in ordine\35.jpg

ID=34

SCALE=0.087005

LM=6

1102.00000 1474.00000

1124.00000 1471.00000

1130.00000 1428.00000

1171.00000 1411.00000

1196.00000 1428.00000

1190.00000 1459.00000

CURVES=3

POINTS=20

1123.00000 1467.00000

1126.00000 1460.00000

1128.00000 1452.00000

1127.00000 1445.00000

1128.00000 1437.00000

1129.00000 1429.00000

1135.00000 1424.00000

1139.00000 1418.00000

1145.00000 1413.00000

1151.00000 1409.00000

1159.00000 1407.00000

1167.00000 1405.00000

1174.00000 1405.00000

1182.00000 1408.00000

1189.00000 1411.00000

1195.00000 1414.00000

1201.00000 1419.00000

1203.00000 1427.00000

1204.00000 1435.00000

1202.00000 1442.00000

POINTS=5

1123.00000 1469.00000

1123.00000 1480.00000

1116.00000 1486.00000

1106.00000 1482.00000

1104.00000 1472.00000

POINTS=5

1201.00000 1442.00000

1194.00000 1444.00000

1188.00000 1447.00000

1186.00000 1452.00000

1186.00000 1459.00000

IMAGE=C:\Users\Pc\Desktop\tutte rx in ordine\36.jpg

ID=35

SCALE=0.089797

LM=6

312.00000 503.00000

330.00000 500.00000

324.00000 485.00000

340.00000 475.00000

357.00000 488.00000

345.00000 493.00000

CURVES=3

POINTS=20

329.00000 499.00000

328.00000 496.00000

326.00000 493.00000

326.00000 490.00000

325.00000 487.00000

326.00000 483.00000

328.00000 481.00000

330.00000 478.00000

332.00000 475.00000

335.00000 475.00000

338.00000 473.00000

341.00000 473.00000

344.00000 473.00000

348.00000 473.00000

351.00000 475.00000

353.00000 477.00000

356.00000 479.00000

358.00000 481.00000

359.00000 484.00000

358.00000 487.00000

POINTS=5

329.00000 499.00000

326.00000 505.00000

321.00000 509.00000

315.00000 506.00000

313.00000 501.00000

POINTS=5

356.00000 486.00000

351.00000 487.00000

346.00000 488.00000

343.00000 491.00000

340.00000 495.00000

IMAGE=C:\Users\Pc\Desktop\tutte rx in ordine\37.bmp

ID=36

SCALE=0.266572

LM=6

1318.00000 1172.00000

1350.00000 1165.00000

1322.00000 1122.00000

1360.00000 1101.00000

1403.00000 1130.00000

1371.00000 1151.00000

CURVES=3

POINTS=20

1348.00000 1163.00000

1340.00000 1157.00000

1332.00000 1151.00000

1323.00000 1147.00000

1318.00000 1139.00000

1317.00000 1129.00000

1320.00000 1120.00000

1326.00000 1112.00000

1332.00000 1104.00000

1338.00000 1097.00000

1347.00000 1092.00000

1356.00000 1091.00000

1366.00000 1091.00000

1375.00000 1094.00000

1385.00000 1097.00000

1393.00000 1102.00000

1398.00000 1110.00000

1402.00000 1119.00000

1405.00000 1128.00000

1405.00000 1138.00000

POINTS=5

1352.00000 1163.00000

1355.00000 1175.00000

1344.00000 1179.00000

1332.00000 1180.00000

1322.00000 1175.00000

POINTS=5

1409.00000 1135.00000

1398.00000 1138.00000

1386.00000 1140.00000

1375.00000 1145.00000

1371.00000 1155.00000

IMAGE=C:\Users\Pc\Desktop\tutte rx in ordine\38.JPG

ID=37

SCALE=0.104703

LM=6

1455.00000 1018.00000

1481.00000 1016.00000

1471.00000 990.00000

1502.00000 964.00000

1539.00000 1004.00000

1521.00000 1023.00000

CURVES=3

POINTS=20

1483.00000 1014.00000

1476.00000 1010.00000

1473.00000 1004.00000

1470.00000 997.00000

1469.00000 989.00000

1471.00000 982.00000

1476.00000 976.00000

1482.00000 972.00000

1488.00000 968.00000

1494.00000 963.00000

1502.00000 962.00000

1509.00000 963.00000

1517.00000 964.00000

1524.00000 966.00000

1528.00000 972.00000

1531.00000 979.00000

1534.00000 986.00000

1538.00000 992.00000

1540.00000 999.00000

1540.00000 1007.00000

POINTS=5

1484.00000 1020.00000

1479.00000 1027.00000

1470.00000 1028.00000

1463.00000 1025.00000

1457.00000 1018.00000

POINTS=5

1539.00000 1011.00000

1531.00000 1012.00000

1524.00000 1016.00000

1519.00000 1022.00000

1517.00000 1030.00000

IMAGE=C:\Users\Pc\Desktop\tutte rx in ordine\39.JPG

ID=38

SCALE=0.135097

LM=6

1534.00000 1609.00000

1572.00000 1600.00000

1556.00000 1559.00000

1604.00000 1525.00000

1666.00000 1586.00000

1631.00000 1613.00000

CURVES=3

POINTS=20

1566.00000 1596.00000

1560.00000 1586.00000

1554.00000 1575.00000

1547.00000 1566.00000

1548.00000 1554.00000

1553.00000 1543.00000

1561.00000 1534.00000

1571.00000 1528.00000

1582.00000 1523.00000

1595.00000 1522.00000

1607.00000 1522.00000

1619.00000 1525.00000

1630.00000 1530.00000

1642.00000 1534.00000

1652.00000 1542.00000

1661.00000 1550.00000

1666.00000 1561.00000

1670.00000 1573.00000

1673.00000 1585.00000

1670.00000 1597.00000

POINTS=5

1572.00000 1602.00000

1569.00000 1614.00000

1559.00000 1620.00000

1547.00000 1618.00000

1537.00000 1609.00000

POINTS=5

1668.00000 1599.00000

1654.00000 1598.00000

1641.00000 1597.00000

1629.00000 1603.00000

1626.00000 1616.00000

IMAGE=C:\Users\Pc\Desktop\tutte rx in ordine\40.jpg

ID=39

SCALE=0.090680

LM=6

1548.00000 1664.00000

1586.00000 1654.00000

1560.00000 1594.00000

1607.00000 1556.00000

1660.00000 1610.00000

1625.00000 1652.00000

CURVES=3

POINTS=20

1584.00000 1648.00000

1577.00000 1638.00000

1568.00000 1628.00000

1564.00000 1615.00000

1561.00000 1602.00000

1561.00000 1589.00000

1566.00000 1577.00000

1575.00000 1567.00000

1584.00000 1559.00000

1597.00000 1555.00000

1609.00000 1552.00000

1622.00000 1554.00000

1634.00000 1559.00000

1644.00000 1566.00000

1652.00000 1577.00000

1657.00000 1589.00000

1663.00000 1601.00000

1665.00000 1614.00000

1666.00000 1627.00000

1664.00000 1640.00000

POINTS=5

1586.00000 1652.00000

1590.00000 1670.00000

1579.00000 1684.00000

1563.00000 1677.00000

1555.00000 1660.00000

POINTS=5

1663.00000 1636.00000

1649.00000 1640.00000

1635.00000 1639.00000

1623.00000 1644.00000

1621.00000 1656.00000

IMAGE=C:\Users\Pc\Desktop\tutte rx in ordine\41.jpg

ID=40

SCALE=0.089533

LM=6

627.00000 1268.00000

668.00000 1259.00000

648.00000 1229.00000

702.00000 1195.00000

756.00000 1252.00000

724.00000 1297.00000

CURVES=3

POINTS=20

661.00000 1253.00000

656.00000 1243.00000

653.00000 1233.00000

653.00000 1223.00000

655.00000 1213.00000

664.00000 1205.00000

674.00000 1203.00000

683.00000 1200.00000

694.00000 1198.00000

704.00000 1194.00000

714.00000 1195.00000

725.00000 1197.00000

733.00000 1203.00000

741.00000 1209.00000

747.00000 1217.00000

751.00000 1226.00000

755.00000 1236.00000

756.00000 1247.00000

756.00000 1258.00000

753.00000 1268.00000

POINTS=5

668.00000 1258.00000

665.00000 1271.00000

655.00000 1280.00000

642.00000 1277.00000

634.00000 1267.00000

POINTS=5

748.00000 1273.00000

737.00000 1276.00000

727.00000 1281.00000

721.00000 1290.00000

721.00000 1301.00000

IMAGE=C:\Users\Pc\Desktop\tutte rx in ordine\42.JPG

ID=41

SCALE=0.002187

LM=6

1016.00000 1581.00000

1043.00000 1576.00000

1040.00000 1540.00000

1093.00000 1516.00000

1124.00000 1540.00000

1106.00000 1579.00000

CURVES=3

POINTS=20

1041.00000 1576.00000

1039.00000 1567.00000

1038.00000 1559.00000

1037.00000 1550.00000

1037.00000 1541.00000

1040.00000 1533.00000

1046.00000 1526.00000

1051.00000 1520.00000

1058.00000 1514.00000

1067.00000 1511.00000

1075.00000 1508.00000

1084.00000 1508.00000

1092.00000 1508.00000

1101.00000 1509.00000

1110.00000 1510.00000

1117.00000 1515.00000

1123.00000 1521.00000

1127.00000 1528.00000

1128.00000 1537.00000

1128.00000 1546.00000

POINTS=5

1045.00000 1579.00000

1044.00000 1592.00000

1033.00000 1597.00000

1022.00000 1591.00000

1018.00000 1578.00000

POINTS=5

1128.00000 1546.00000

1124.00000 1557.00000

1116.00000 1565.00000

1105.00000 1569.00000

1102.00000 1580.00000

IMAGE=C:\Users\Pc\Desktop\tutte rx in ordine\43.jpeg

ID=42

SCALE=0.083490

LM=6

1552.00000 1539.00000

1579.00000 1528.00000

1578.00000 1487.00000

1620.00000 1473.00000

1667.00000 1536.00000

1648.00000 1570.00000

CURVES=3

POINTS=20

1579.00000 1530.00000

1578.00000 1520.00000

1575.00000 1510.00000

1575.00000 1499.00000

1576.00000 1489.00000

1581.00000 1479.00000

1588.00000 1472.00000

1597.00000 1466.00000

1608.00000 1465.00000

1618.00000 1464.00000

1629.00000 1465.00000

1638.00000 1470.00000

1647.00000 1476.00000

1654.00000 1483.00000

1661.00000 1491.00000

1663.00000 1501.00000

1666.00000 1512.00000

1666.00000 1522.00000

1663.00000 1532.00000

1664.00000 1543.00000

POINTS=5

1583.00000 1535.00000

1578.00000 1544.00000

1569.00000 1549.00000

1559.00000 1546.00000

1554.00000 1537.00000

POINTS=5

1663.00000 1545.00000

1656.00000 1550.00000

1651.00000 1557.00000

1647.00000 1565.00000

1646.00000 1574.00000

IMAGE=C:\Users\Pc\Desktop\tutte rx in ordine\44.jpg

ID=43

SCALE=0.091115

LM=6

1326.00000 1228.00000

1348.00000 1228.00000

1329.00000 1181.00000

1373.00000 1146.00000

1419.00000 1213.00000

1380.00000 1240.00000

CURVES=3

POINTS=20

1349.00000 1228.00000

1343.00000 1218.00000

1340.00000 1210.00000

1333.00000 1200.00000

1331.00000 1194.00000

1327.00000 1183.00000

1330.00000 1172.00000

1336.00000 1163.00000

1346.00000 1158.00000

1356.00000 1151.00000

1366.00000 1146.00000

1378.00000 1146.00000

1389.00000 1151.00000

1397.00000 1160.00000

1405.00000 1167.00000

1410.00000 1177.00000

1415.00000 1188.00000

1416.00000 1200.00000

1419.00000 1211.00000

1419.00000 1223.00000

POINTS=5

1349.00000 1228.00000

1349.00000 1239.00000

1341.00000 1245.00000

1333.00000 1238.00000

1328.00000 1228.00000

POINTS=5

1419.00000 1223.00000

1406.00000 1224.00000

1393.00000 1224.00000

1383.00000 1231.00000

1378.00000 1242.00000

IMAGE=C:\Users\Pc\Desktop\tutte rx in ordine\45.JPG

ID=44

SCALE=0.114611

LM=6

142.00000 216.00000

147.00000 214.00000

144.00000 208.00000

150.00000 205.00000

159.00000 212.00000

155.00000 218.00000

CURVES=3

POINTS=20

148.00000 217.00000

147.00000 216.00000

146.00000 214.00000

145.00000 213.00000

145.00000 211.00000

145.00000 209.00000

145.00000 208.00000

147.00000 207.00000

147.00000 206.00000

149.00000 206.00000

150.00000 205.00000

152.00000 205.00000

154.00000 206.00000

155.00000 207.00000

157.00000 208.00000

158.00000 209.00000

159.00000 210.00000

160.00000 211.00000

160.00000 213.00000

159.00000 214.00000

POINTS=5

149.00000 219.00000

147.00000 221.00000

145.00000 221.00000

143.00000 219.00000

142.00000 217.00000

POINTS=5

158.00000 215.00000

156.00000 215.00000

154.00000 215.00000

153.00000 216.00000

153.00000 218.00000

IMAGE=C:\Users\Pc\Desktop\tutte rx in ordine\46.PNG

ID=45

SCALE=0.808736

LM=6

101.00000 154.00000

104.00000 154.00000

102.00000 150.00000

108.00000 147.00000

112.00000 152.00000

110.00000 156.00000

CURVES=3

POINTS=20

104.00000 155.00000

104.00000 154.00000

104.00000 153.00000

103.00000 152.00000

103.00000 151.00000

103.00000 150.00000

103.00000 149.00000

104.00000 148.00000

105.00000 148.00000

107.00000 148.00000

108.00000 148.00000

109.00000 148.00000

110.00000 148.00000

110.00000 149.00000

111.00000 150.00000

112.00000 151.00000

112.00000 152.00000

112.00000 153.00000

111.00000 154.00000

110.00000 155.00000

POINTS=5

104.00000 155.00000

104.00000 156.00000

102.00000 156.00000

101.00000 155.00000

101.00000 154.00000

POINTS=5

110.00000 155.00000

109.00000 155.00000

108.00000 155.00000

108.00000 156.00000

107.00000 157.00000

IMAGE=C:\Users\Pc\Desktop\tutte rx in ordine\47.PNG

ID=46

SCALE=0.995893

LM=6

1232.00000 1404.00000

1279.00000 1397.00000

1236.00000 1327.00000

1285.00000 1296.00000

1361.00000 1350.00000

1299.00000 1381.00000

CURVES=3

POINTS=20

1276.00000 1399.00000

1266.00000 1388.00000

1260.00000 1375.00000

1252.00000 1363.00000

1246.00000 1351.00000

1240.00000 1338.00000

1242.00000 1325.00000

1250.00000 1313.00000

1259.00000 1303.00000

1269.00000 1294.00000

1281.00000 1287.00000

1295.00000 1287.00000

1309.00000 1291.00000

1322.00000 1297.00000

1333.00000 1306.00000

1344.00000 1315.00000

1351.00000 1328.00000

1357.00000 1340.00000

1363.00000 1353.00000

1369.00000 1366.00000

POINTS=5

1278.00000 1403.00000

1279.00000 1420.00000

1262.00000 1424.00000

1245.00000 1417.00000

1231.00000 1404.00000

POINTS=5

1370.00000 1368.00000

1353.00000 1367.00000

1336.00000 1368.00000

1320.00000 1373.00000

1310.00000 1386.00000

IMAGE=C:\Users\Pc\Desktop\tutte rx in ordine\48.JPG

ID=47

SCALE=0.073800

LM=6

1018.00000 1572.00000

1054.00000 1572.00000

1053.00000 1521.00000

1101.00000 1508.00000

1142.00000 1544.00000

1109.00000 1589.00000

CURVES=3

POINTS=20

1053.00000 1572.00000

1051.00000 1562.00000

1051.00000 1553.00000

1051.00000 1543.00000

1051.00000 1533.00000

1055.00000 1524.00000

1063.00000 1519.00000

1072.00000 1514.00000

1078.00000 1510.00000

1088.00000 1508.00000

1098.00000 1506.00000

1107.00000 1506.00000

1117.00000 1509.00000

1125.00000 1511.00000

1132.00000 1518.00000

1137.00000 1526.00000

1138.00000 1535.00000

1142.00000 1544.00000

1144.00000 1554.00000

1140.00000 1561.00000

POINTS=5

1054.00000 1577.00000

1061.00000 1598.00000

1046.00000 1607.00000

1033.00000 1593.00000

1023.00000 1574.00000

POINTS=5

1139.00000 1561.00000

1126.00000 1563.00000

1114.00000 1572.00000

1107.00000 1585.00000

1106.00000 1600.00000

IMAGE=C:\Users\Pc\Desktop\tutte rx in ordine\49.jpeg

ID=48

SCALE=0.107018

LM=6

1506.00000 1172.00000

1555.00000 1169.00000

1557.00000 1129.00000

1592.00000 1107.00000

1628.00000 1141.00000

1606.00000 1170.00000

CURVES=3

POINTS=20

1555.00000 1173.00000

1552.00000 1166.00000

1549.00000 1158.00000

1548.00000 1150.00000

1549.00000 1142.00000

1551.00000 1135.00000

1554.00000 1128.00000

1559.00000 1122.00000

1566.00000 1117.00000

1573.00000 1114.00000

1580.00000 1110.00000

1588.00000 1109.00000

1596.00000 1109.00000

1602.00000 1114.00000

1608.00000 1118.00000

1614.00000 1122.00000

1620.00000 1128.00000

1624.00000 1134.00000

1625.00000 1142.00000

1625.00000 1150.00000

POINTS=5

1554.00000 1174.00000

1543.00000 1195.00000

1526.00000 1211.00000

1515.00000 1191.00000

1506.00000 1170.00000

POINTS=5

1625.00000 1150.00000

1616.00000 1153.00000

1608.00000 1159.00000

1601.00000 1165.00000

1598.00000 1174.00000

IMAGE=C:\Users\Pc\Desktop\tutte rx in ordine\50.JPG

ID=49

SCALE=0.112796

LM=6

1757.00000 1102.00000

1787.00000 1096.00000

1770.00000 1066.00000

1803.00000 1045.00000

1842.00000 1066.00000

1823.00000 1091.00000

CURVES=3

POINTS=20

1785.00000 1096.00000

1782.00000 1088.00000

1778.00000 1082.00000

1774.00000 1075.00000

1771.00000 1067.00000

1767.00000 1060.00000

1767.00000 1051.00000

1772.00000 1047.00000

1779.00000 1043.00000

1786.00000 1041.00000

1794.00000 1039.00000

1802.00000 1038.00000

1810.00000 1038.00000

1818.00000 1040.00000

1825.00000 1044.00000

1831.00000 1049.00000

1837.00000 1054.00000

1842.00000 1061.00000

1845.00000 1069.00000

1846.00000 1077.00000

POINTS=5

1787.00000 1100.00000

1783.00000 1107.00000

1775.00000 1111.00000

1767.00000 1109.00000

1761.00000 1104.00000

POINTS=5

1844.00000 1076.00000

1835.00000 1077.00000

1827.00000 1081.00000

1819.00000 1085.00000

1818.00000 1094.00000

IMAGE=C:\Users\Pc\Desktop\tutte rx in ordine\51.JPG

ID=50

SCALE=0.100748

LM=6

139.00000 230.00000

148.00000 228.00000

140.00000 220.00000

153.00000 215.00000

166.00000 224.00000

159.00000 233.00000

CURVES=3

POINTS=20

148.00000 229.00000

146.00000 227.00000

144.00000 226.00000

142.00000 224.00000

141.00000 223.00000

140.00000 220.00000

141.00000 218.00000

143.00000 217.00000

145.00000 216.00000

147.00000 216.00000

149.00000 215.00000

151.00000 215.00000

154.00000 215.00000

156.00000 216.00000

158.00000 217.00000

160.00000 218.00000

162.00000 220.00000

164.00000 222.00000

164.00000 224.00000

164.00000 227.00000

POINTS=5

148.00000 230.00000

147.00000 234.00000

143.00000 235.00000

141.00000 232.00000

139.00000 229.00000

POINTS=5

164.00000 227.00000

161.00000 228.00000

159.00000 228.00000

157.00000 230.00000

157.00000 233.00000

IMAGE=C:\Users\Pc\Desktop\tutte rx in ordine\52.PNG

ID=51

SCALE=0.495415

LM=6

837.00000 1521.00000

870.00000 1512.00000

877.00000 1468.00000

941.00000 1437.00000

988.00000 1489.00000

934.00000 1514.00000

CURVES=3

POINTS=20

869.00000 1505.00000

867.00000 1494.00000

868.00000 1483.00000

873.00000 1473.00000

879.00000 1464.00000

887.00000 1457.00000

895.00000 1450.00000

905.00000 1444.00000

915.00000 1439.00000

925.00000 1435.00000

937.00000 1435.00000

947.00000 1437.00000

958.00000 1441.00000

968.00000 1446.00000

976.00000 1453.00000

980.00000 1464.00000

983.00000 1474.00000

987.00000 1485.00000

987.00000 1496.00000

981.00000 1505.00000

POINTS=5

869.00000 1517.00000

861.00000 1523.00000

849.00000 1526.00000

841.00000 1520.00000

839.00000 1508.00000

POINTS=5

978.00000 1507.00000

964.00000 1503.00000

951.00000 1498.00000

936.00000 1499.00000

931.00000 1512.00000

IMAGE=C:\Users\Pc\Desktop\tutte rx in ordine\53.jpg

ID=52

SCALE=0.087711

LM=6

994.00000 1525.00000

1035.00000 1525.00000

1035.00000 1492.00000

1072.00000 1469.00000

1109.00000 1525.00000

1072.00000 1540.00000

CURVES=3

POINTS=20

1035.00000 1523.00000

1034.00000 1515.00000

1034.00000 1506.00000

1034.00000 1497.00000

1039.00000 1491.00000

1042.00000 1483.00000

1048.00000 1476.00000

1055.00000 1471.00000

1064.00000 1468.00000

1072.00000 1467.00000

1081.00000 1466.00000

1089.00000 1468.00000

1096.00000 1473.00000

1102.00000 1479.00000

1107.00000 1486.00000

1110.00000 1494.00000

1113.00000 1502.00000

1115.00000 1510.00000

1115.00000 1519.00000

1112.00000 1527.00000

POINTS=5

1034.00000 1529.00000

1026.00000 1538.00000

1014.00000 1540.00000

1005.00000 1532.00000

1005.00000 1520.00000

POINTS=5

1112.00000 1533.00000

1101.00000 1532.00000

1090.00000 1529.00000

1079.00000 1529.00000

1072.00000 1537.00000

IMAGE=C:\Users\Pc\Desktop\tutte rx in ordine\54.jpg

ID=53

SCALE=0.090479

LM=6

946.00000 1605.00000

969.00000 1607.00000

973.00000 1563.00000

1030.00000 1540.00000

1089.00000 1605.00000

1053.00000 1643.00000

CURVES=3

POINTS=20

964.00000 1602.00000

963.00000 1590.00000

964.00000 1578.00000

970.00000 1568.00000

976.00000 1558.00000

987.00000 1554.00000

996.00000 1549.00000

1008.00000 1546.00000

1020.00000 1544.00000

1032.00000 1543.00000

1044.00000 1543.00000

1055.00000 1546.00000

1065.00000 1553.00000

1075.00000 1559.00000

1081.00000 1569.00000

1086.00000 1581.00000

1087.00000 1592.00000

1088.00000 1604.00000

1088.00000 1616.00000

1083.00000 1626.00000

POINTS=5

970.00000 1607.00000

969.00000 1617.00000

959.00000 1620.00000

950.00000 1616.00000

947.00000 1606.00000

POINTS=5

1077.00000 1627.00000

1068.00000 1628.00000

1060.00000 1632.00000

1054.00000 1636.00000

1050.00000 1643.00000

IMAGE=C:\Users\Pc\Desktop\tutte rx in ordine\55.jpg

ID=54

SCALE=0.085322

LM=6

311.00000 458.00000

324.00000 457.00000

323.00000 442.00000

335.00000 433.00000

349.00000 445.00000

337.00000 460.00000

CURVES=3

POINTS=20

323.00000 457.00000

323.00000 454.00000

322.00000 450.00000

322.00000 447.00000

321.00000 444.00000

319.00000 441.00000

322.00000 439.00000

324.00000 437.00000

326.00000 435.00000

329.00000 433.00000

333.00000 433.00000

336.00000 434.00000

339.00000 435.00000

342.00000 436.00000

344.00000 439.00000

347.00000 441.00000

348.00000 443.00000

350.00000 446.00000

351.00000 450.00000

351.00000 453.00000

POINTS=5

324.00000 457.00000

325.00000 462.00000

320.00000 467.00000

316.00000 464.00000

313.00000 459.00000

POINTS=5

350.00000 453.00000

346.00000 454.00000

342.00000 456.00000

339.00000 458.00000

338.00000 461.00000

IMAGE=C:\Users\Pc\Desktop\tutte rx in ordine\56.bmp

ID=55

SCALE=0.264848

LM=6

1075.00000 1555.00000

1108.00000 1550.00000

1087.00000 1515.00000

1152.00000 1472.00000

1193.00000 1519.00000

1157.00000 1557.00000

CURVES=3

POINTS=20

1107.00000 1547.00000

1100.00000 1540.00000

1094.00000 1533.00000

1090.00000 1524.00000

1089.00000 1514.00000

1091.00000 1504.00000

1098.00000 1497.00000

1106.00000 1491.00000

1113.00000 1485.00000

1120.00000 1480.00000

1130.00000 1477.00000

1140.00000 1476.00000

1150.00000 1476.00000

1159.00000 1479.00000

1168.00000 1483.00000

1176.00000 1488.00000

1185.00000 1493.00000

1190.00000 1501.00000

1193.00000 1510.00000

1193.00000 1520.00000

POINTS=5

1110.00000 1554.00000

1105.00000 1562.00000

1095.00000 1566.00000

1087.00000 1561.00000

1080.00000 1554.00000

POINTS=5

1191.00000 1520.00000

1178.00000 1527.00000

1166.00000 1535.00000

1154.00000 1544.00000

1149.00000 1557.00000

IMAGE=C:\Users\Pc\Desktop\tutte rx in ordine\57.jpg

ID=56

SCALE=0.088940

LM=6

940.00000 1489.00000

980.00000 1485.00000

954.00000 1430.00000

1011.00000 1404.00000

1088.00000 1461.00000

1070.00000 1504.00000

CURVES=3

POINTS=20

979.00000 1488.00000

971.00000 1476.00000

961.00000 1464.00000

956.00000 1450.00000

952.00000 1436.00000

952.00000 1421.00000

959.00000 1409.00000

971.00000 1401.00000

984.00000 1396.00000

999.00000 1392.00000

1013.00000 1392.00000

1026.00000 1394.00000

1039.00000 1400.00000

1053.00000 1405.00000

1064.00000 1414.00000

1073.00000 1426.00000

1080.00000 1439.00000

1086.00000 1453.00000

1092.00000 1467.00000

1092.00000 1481.00000

POINTS=5

983.00000 1490.00000

976.00000 1504.00000

962.00000 1510.00000

950.00000 1503.00000

941.00000 1491.00000

POINTS=5

1092.00000 1482.00000

1082.00000 1487.00000

1072.00000 1492.00000

1062.00000 1497.00000

1058.00000 1508.00000

IMAGE=C:\Users\Pc\Desktop\tutte rx in ordine\58.jpg

ID=57

SCALE=0.094686

LM=6

1061.00000 1524.00000

1102.00000 1524.00000

1100.00000 1491.00000

1157.00000 1460.00000

1190.00000 1497.00000

1154.00000 1527.00000

CURVES=3

POINTS=20

1102.00000 1517.00000

1102.00000 1507.00000

1101.00000 1498.00000

1101.00000 1488.00000

1105.00000 1480.00000

1110.00000 1471.00000

1115.00000 1464.00000

1124.00000 1460.00000

1132.00000 1455.00000

1141.00000 1453.00000

1151.00000 1453.00000

1160.00000 1451.00000

1168.00000 1454.00000

1177.00000 1457.00000

1185.00000 1460.00000

1190.00000 1469.00000

1194.00000 1477.00000

1194.00000 1487.00000

1194.00000 1496.00000

1194.00000 1506.00000

POINTS=5

1101.00000 1526.00000

1095.00000 1535.00000

1084.00000 1536.00000

1073.00000 1531.00000

1064.00000 1524.00000

POINTS=5

1194.00000 1513.00000

1183.00000 1516.00000

1171.00000 1517.00000

1159.00000 1517.00000

1154.00000 1526.00000

IMAGE=C:\Users\Pc\Desktop\tutte rx in ordine\59.jpg

ID=58

SCALE=0.085870

LM=6

2022.00000 1882.00000

2071.00000 1881.00000

2065.00000 1826.00000

2125.00000 1811.00000

2169.00000 1854.00000

2116.00000 1889.00000

CURVES=3

POINTS=20

2065.00000 1877.00000

2062.00000 1867.00000

2058.00000 1858.00000

2057.00000 1847.00000

2060.00000 1838.00000

2065.00000 1828.00000

2072.00000 1821.00000

2079.00000 1813.00000

2089.00000 1809.00000

2099.00000 1806.00000

2109.00000 1805.00000

2119.00000 1805.00000

2130.00000 1806.00000

2140.00000 1808.00000

2150.00000 1812.00000

2158.00000 1818.00000

2164.00000 1827.00000

2167.00000 1836.00000

2170.00000 1847.00000

2170.00000 1857.00000

POINTS=5

2066.00000 1879.00000

2061.00000 1890.00000

2049.00000 1892.00000

2037.00000 1890.00000

2027.00000 1883.00000

POINTS=5

2170.00000 1862.00000

2156.00000 1870.00000

2140.00000 1874.00000

2123.00000 1875.00000

2118.00000 1891.00000

IMAGE=C:\Users\Pc\Desktop\tutte rx in ordine\60.JPG

ID=59

SCALE=0.085016

LM=6

995.00000 1552.00000

1051.00000 1538.00000

1026.00000 1479.00000

1073.00000 1451.00000

1129.00000 1491.00000

1086.00000 1531.00000

CURVES=3

POINTS=20

1040.00000 1531.00000

1032.00000 1522.00000

1027.00000 1512.00000

1023.00000 1500.00000

1023.00000 1489.00000

1026.00000 1477.00000

1030.00000 1466.00000

1038.00000 1458.00000

1048.00000 1451.00000

1059.00000 1447.00000

1071.00000 1446.00000

1083.00000 1446.00000

1095.00000 1446.00000

1106.00000 1449.00000

1117.00000 1454.00000

1125.00000 1463.00000

1131.00000 1473.00000

1136.00000 1484.00000

1136.00000 1495.00000

1134.00000 1507.00000

POINTS=5

1054.00000 1538.00000

1057.00000 1561.00000

1036.00000 1573.00000

1013.00000 1566.00000

997.00000 1547.00000

POINTS=5

1136.00000 1515.00000

1117.00000 1512.00000

1099.00000 1512.00000

1083.00000 1520.00000

1080.00000 1538.00000

IMAGE=C:\Users\Pc\Desktop\tutte rx in ordine\61.jpg

ID=60

SCALE=0.085608

LM=6

688.00000 408.00000

696.00000 407.00000

693.00000 396.00000

701.00000 392.00000

713.00000 402.00000

704.00000 409.00000

CURVES=3

POINTS=20

695.00000 408.00000

693.00000 407.00000

693.00000 405.00000

692.00000 403.00000

691.00000 400.00000

692.00000 398.00000

693.00000 397.00000

694.00000 395.00000

696.00000 393.00000

698.00000 392.00000

700.00000 392.00000

702.00000 392.00000

705.00000 392.00000

707.00000 393.00000

708.00000 394.00000

710.00000 395.00000

711.00000 397.00000

711.00000 399.00000

712.00000 402.00000

712.00000 404.00000

POINTS=5

696.00000 409.00000

694.00000 411.00000

691.00000 412.00000

688.00000 409.00000

688.00000 406.00000

POINTS=5

712.00000 404.00000

710.00000 406.00000

706.00000 407.00000

704.00000 408.00000

702.00000 411.00000

IMAGE=C:\Users\Pc\Desktop\tutte rx in ordine\62.jpg

ID=61

SCALE=0.392081

LM=6

1501.00000 1415.00000

1523.00000 1413.00000

1515.00000 1367.00000

1558.00000 1338.00000

1596.00000 1363.00000

1575.00000 1389.00000

CURVES=3

POINTS=20

1523.00000 1405.00000

1518.00000 1398.00000

1514.00000 1390.00000

1512.00000 1381.00000

1510.00000 1373.00000

1511.00000 1364.00000

1516.00000 1358.00000

1522.00000 1351.00000

1528.00000 1346.00000

1536.00000 1342.00000

1543.00000 1339.00000

1551.00000 1335.00000

1560.00000 1335.00000

1568.00000 1336.00000

1577.00000 1339.00000

1585.00000 1342.00000

1591.00000 1349.00000

1596.00000 1355.00000

1600.00000 1363.00000

1600.00000 1372.00000

POINTS=5

1523.00000 1415.00000

1520.00000 1425.00000

1512.00000 1432.00000

1505.00000 1425.00000

1500.00000 1415.00000

POINTS=5

1601.00000 1369.00000

1591.00000 1374.00000

1580.00000 1377.00000

1573.00000 1386.00000

1573.00000 1397.00000

IMAGE=C:\Users\Pc\Desktop\tutte rx in ordine\63.tif

ID=62

SCALE=0.091742

LM=6

1476.00000 1367.00000

1505.00000 1361.00000

1495.00000 1312.00000

1552.00000 1283.00000

1595.00000 1309.00000

1562.00000 1345.00000

CURVES=3

POINTS=20

1499.00000 1358.00000

1498.00000 1347.00000

1492.00000 1338.00000

1488.00000 1328.00000

1489.00000 1317.00000

1491.00000 1306.00000

1497.00000 1298.00000

1502.00000 1288.00000

1511.00000 1281.00000

1521.00000 1279.00000

1532.00000 1276.00000

1542.00000 1273.00000

1553.00000 1273.00000

1564.00000 1274.00000

1574.00000 1278.00000

1583.00000 1284.00000

1593.00000 1289.00000

1601.00000 1296.00000

1608.00000 1304.00000

1609.00000 1315.00000

POINTS=5

1507.00000 1366.00000

1510.00000 1380.00000

1499.00000 1389.00000

1487.00000 1380.00000

1479.00000 1366.00000

POINTS=5

1606.00000 1325.00000

1591.00000 1323.00000

1576.00000 1327.00000

1564.00000 1336.00000

1560.00000 1350.00000

IMAGE=C:\Users\Pc\Desktop\tutte rx in ordine\64.JPG

ID=63

SCALE=0.002380

LM=6

611.00000 664.00000

624.00000 663.00000

630.00000 642.00000

654.00000 630.00000

682.00000 651.00000

659.00000 672.00000

CURVES=3

POINTS=20

629.00000 663.00000

628.00000 658.00000

628.00000 653.00000

628.00000 648.00000

627.00000 643.00000

630.00000 639.00000

633.00000 636.00000

638.00000 634.00000

643.00000 633.00000

647.00000 632.00000

653.00000 632.00000

658.00000 632.00000

663.00000 632.00000

667.00000 633.00000

672.00000 636.00000

675.00000 639.00000

679.00000 643.00000

681.00000 647.00000

682.00000 652.00000

682.00000 657.00000

POINTS=5

630.00000 669.00000

629.00000 678.00000

620.00000 679.00000

613.00000 672.00000

610.00000 664.00000

POINTS=5

682.00000 660.00000

674.00000 663.00000

665.00000 662.00000

658.00000 665.00000

654.00000 673.00000

IMAGE=C:\Users\Pc\Desktop\tutte rx in ordine\65.jpg

ID=64

SCALE=0.149216

LM=6

708.00000 599.00000

725.00000 596.00000

711.00000 580.00000

730.00000 566.00000

751.00000 596.00000

739.00000 606.00000

CURVES=3

POINTS=20

726.00000 599.00000

722.00000 595.00000

718.00000 591.00000

715.00000 587.00000

712.00000 583.00000

711.00000 578.00000

711.00000 572.00000

714.00000 568.00000

719.00000 566.00000

723.00000 564.00000

729.00000 565.00000

734.00000 565.00000

739.00000 566.00000

743.00000 570.00000

747.00000 572.00000

749.00000 577.00000

751.00000 582.00000

752.00000 587.00000

752.00000 592.00000

750.00000 597.00000

POINTS=5

725.00000 598.00000

725.00000 603.00000

722.00000 606.00000

717.00000 606.00000

713.00000 604.00000

POINTS=5

750.00000 598.00000

746.00000 600.00000

743.00000 603.00000

739.00000 603.00000

736.00000 606.00000

IMAGE=C:\Users\Pc\Desktop\tutte rx in ordine\66.jpg

ID=65

SCALE=0.180710

LM=6

760.00000 534.00000

778.00000 531.00000

772.00000 505.00000

787.00000 489.00000

814.00000 511.00000

800.00000 530.00000

CURVES=3

POINTS=20

776.00000 528.00000

775.00000 522.00000

772.00000 517.00000

771.00000 511.00000

769.00000 506.00000

770.00000 500.00000

773.00000 495.00000

776.00000 491.00000

780.00000 486.00000

786.00000 486.00000

792.00000 485.00000

798.00000 485.00000

803.00000 488.00000

807.00000 492.00000

811.00000 496.00000

814.00000 500.00000

814.00000 506.00000

814.00000 513.00000

814.00000 519.00000

812.00000 524.00000

POINTS=5

774.00000 529.00000

773.00000 536.00000

769.00000 542.00000

764.00000 545.00000

761.00000 540.00000

POINTS=5

809.00000 524.00000

805.00000 524.00000

802.00000 525.00000

799.00000 528.00000

798.00000 531.00000

IMAGE=C:\Users\Pc\Desktop\tutte rx in ordine\67.jpg

ID=66

SCALE=0.188289

LM=6

638.00000 564.00000

654.00000 564.00000

651.00000 544.00000

673.00000 532.00000

697.00000 557.00000

673.00000 575.00000

CURVES=3

POINTS=20

652.00000 566.00000

651.00000 561.00000

649.00000 556.00000

649.00000 551.00000

649.00000 545.00000

651.00000 541.00000

654.00000 536.00000

658.00000 533.00000

663.00000 533.00000

668.00000 532.00000

673.00000 530.00000

678.00000 532.00000

683.00000 534.00000

688.00000 536.00000

692.00000 539.00000

694.00000 544.00000

696.00000 549.00000

699.00000 553.00000

699.00000 559.00000

698.00000 564.00000

POINTS=5

653.00000 570.00000

650.00000 575.00000

644.00000 576.00000

641.00000 571.00000

639.00000 566.00000

POINTS=5

697.00000 566.00000

689.00000 566.00000

681.00000 568.00000

674.00000 571.00000

671.00000 577.00000

IMAGE=C:\Users\Pc\Desktop\tutte rx in ordine\68.jpg

ID=67

SCALE=0.169340

LM=6

309.00000 373.00000

319.00000 371.00000

317.00000 364.00000

324.00000 360.00000

339.00000 366.00000

334.00000 373.00000

CURVES=3

POINTS=20

318.00000 374.00000

317.00000 372.00000

316.00000 370.00000

314.00000 368.00000

314.00000 366.00000

315.00000 364.00000

317.00000 363.00000

318.00000 362.00000

320.00000 360.00000

323.00000 360.00000

325.00000 360.00000

327.00000 360.00000

329.00000 361.00000

332.00000 361.00000

334.00000 362.00000

336.00000 363.00000

338.00000 364.00000

339.00000 366.00000

340.00000 368.00000

340.00000 370.00000

POINTS=5

319.00000 376.00000

317.00000 378.00000

314.00000 378.00000

311.00000 376.00000

310.00000 373.00000

POINTS=5

340.00000 370.00000

337.00000 370.00000

334.00000 371.00000

333.00000 372.00000

332.00000 375.00000

IMAGE=C:\Users\Pc\Desktop\tutte rx in ordine\69.PNG

ID=68

SCALE=0.388276

LM=6

803.00000 536.00000

822.00000 537.00000

815.00000 522.00000

833.00000 509.00000

850.00000 528.00000

839.00000 542.00000

CURVES=3

POINTS=20

823.00000 536.00000

821.00000 532.00000

817.00000 530.00000

815.00000 526.00000

814.00000 522.00000

814.00000 517.00000

814.00000 513.00000

817.00000 510.00000

820.00000 508.00000

824.00000 506.00000

829.00000 506.00000

833.00000 506.00000

837.00000 507.00000

841.00000 508.00000

845.00000 512.00000

847.00000 515.00000

850.00000 518.00000

851.00000 522.00000

850.00000 527.00000

850.00000 531.00000

POINTS=5

823.00000 539.00000

817.00000 545.00000

811.00000 549.00000

805.00000 542.00000

803.00000 534.00000

POINTS=5

850.00000 530.00000

846.00000 534.00000

842.00000 536.00000

837.00000 538.00000

836.00000 543.00000

IMAGE=C:\Users\Pc\Desktop\tutte rx in ordine\70.jpg

ID=69

SCALE=0.194801

LM=6

630.00000 730.00000

656.00000 727.00000

637.00000 705.00000

664.00000 683.00000

707.00000 706.00000

706.00000 735.00000

CURVES=3

POINTS=20

655.00000 727.00000

651.00000 721.00000

646.00000 715.00000

640.00000 710.00000

639.00000 704.00000

638.00000 696.00000

641.00000 690.00000

645.00000 685.00000

652.00000 682.00000

659.00000 681.00000

666.00000 680.00000

674.00000 680.00000

680.00000 682.00000

687.00000 685.00000

694.00000 689.00000

700.00000 691.00000

705.00000 696.00000

709.00000 703.00000

711.00000 710.00000

711.00000 717.00000

POINTS=5

660.00000 727.00000

661.00000 739.00000

651.00000 743.00000

640.00000 737.00000

630.00000 731.00000

POINTS=5

711.00000 718.00000

710.00000 725.00000

703.00000 728.00000

699.00000 734.00000

701.00000 741.00000

IMAGE=C:\Users\Pc\Desktop\tutte rx in ordine\71.jpg

ID=70

SCALE=0.216646

LM=6

796.00000 553.00000

812.00000 549.00000

814.00000 532.00000

844.00000 520.00000

859.00000 536.00000

843.00000 547.00000

CURVES=3

POINTS=20

815.00000 550.00000

813.00000 546.00000

813.00000 542.00000

812.00000 537.00000

813.00000 533.00000

815.00000 529.00000

818.00000 526.00000

821.00000 523.00000

825.00000 521.00000

828.00000 519.00000

833.00000 518.00000

837.00000 518.00000

842.00000 518.00000

846.00000 518.00000

850.00000 521.00000

854.00000 523.00000

857.00000 527.00000

860.00000 530.00000

860.00000 535.00000

861.00000 539.00000

POINTS=5

816.00000 553.00000

817.00000 563.00000

809.00000 568.00000

800.00000 564.00000

796.00000 556.00000

POINTS=5

859.00000 538.00000

853.00000 539.00000

848.00000 541.00000

844.00000 544.00000

844.00000 550.00000

IMAGE=C:\Users\Pc\Desktop\tutte rx in ordine\72.jpg

ID=71

SCALE=0.180710

LM=6

646.00000 637.00000

663.00000 632.00000

653.00000 611.00000

682.00000 591.00000

705.00000 610.00000

693.00000 622.00000

CURVES=3

POINTS=20

661.00000 632.00000

659.00000 627.00000

656.00000 622.00000

654.00000 617.00000

653.00000 612.00000

654.00000 606.00000

656.00000 601.00000

658.00000 596.00000

662.00000 593.00000

667.00000 591.00000

672.00000 590.00000

678.00000 590.00000

683.00000 590.00000

688.00000 591.00000

693.00000 593.00000

698.00000 594.00000

702.00000 599.00000

704.00000 603.00000

705.00000 608.00000

705.00000 614.00000

POINTS=5

663.00000 634.00000

662.00000 640.00000

657.00000 643.00000

651.00000 642.00000

647.00000 637.00000

POINTS=5

705.00000 615.00000

700.00000 615.00000

696.00000 618.00000

692.00000 620.00000

691.00000 624.00000

IMAGE=C:\Users\Pc\Desktop\tutte rx in ordine\73.jpg

ID=72

SCALE=0.158650

LM=6

930.00000 1668.00000

959.00000 1664.00000

940.00000 1633.00000

979.00000 1621.00000

1013.00000 1655.00000

1002.00000 1690.00000

CURVES=3

POINTS=20

959.00000 1667.00000

956.00000 1660.00000

952.00000 1654.00000

946.00000 1649.00000

942.00000 1643.00000

941.00000 1636.00000

945.00000 1630.00000

951.00000 1626.00000

959.00000 1624.00000

966.00000 1622.00000

973.00000 1620.00000

981.00000 1620.00000

988.00000 1622.00000

995.00000 1626.00000

1000.00000 1630.00000

1005.00000 1636.00000

1009.00000 1642.00000

1012.00000 1649.00000

1014.00000 1657.00000

1013.00000 1664.00000

POINTS=5

962.00000 1667.00000

958.00000 1678.00000

947.00000 1681.00000

936.00000 1677.00000

932.00000 1667.00000

POINTS=5

1012.00000 1665.00000

1006.00000 1673.00000

998.00000 1677.00000

991.00000 1684.00000

990.00000 1693.00000

IMAGE=C:\Users\Pc\Desktop\tutte rx in ordine\74.JPG

ID=73

SCALE=0.160852

LM=6

810.00000 570.00000

822.00000 569.00000

824.00000 549.00000

843.00000 542.00000

877.00000 573.00000

859.00000 592.00000

CURVES=3

POINTS=20

822.00000 567.00000

823.00000 562.00000

822.00000 557.00000

822.00000 552.00000

825.00000 548.00000

829.00000 544.00000

833.00000 542.00000

838.00000 541.00000

843.00000 541.00000

848.00000 541.00000

853.00000 541.00000

858.00000 543.00000

863.00000 545.00000

866.00000 549.00000

869.00000 553.00000

872.00000 557.00000

873.00000 561.00000

875.00000 566.00000

876.00000 571.00000

876.00000 576.00000

POINTS=5

822.00000 568.00000

822.00000 573.00000

818.00000 576.00000

813.00000 576.00000

812.00000 572.00000

POINTS=5

876.00000 576.00000

869.00000 579.00000

863.00000 583.00000

857.00000 586.00000

854.00000 592.00000

IMAGE=C:\Users\Pc\Desktop\tutte rx in ordine\75.jpg

ID=74

SCALE=0.166574

LM=6

172.00000 263.00000

176.00000 263.00000

173.00000 256.00000

180.00000 252.00000

187.00000 260.00000

183.00000 267.00000

CURVES=3

POINTS=20

176.00000 264.00000

175.00000 262.00000

174.00000 261.00000

173.00000 259.00000

173.00000 257.00000

173.00000 255.00000

174.00000 253.00000

175.00000 252.00000

176.00000 251.00000

178.00000 251.00000

180.00000 251.00000

182.00000 252.00000

184.00000 253.00000

185.00000 254.00000

187.00000 255.00000

187.00000 257.00000

188.00000 259.00000

188.00000 261.00000

188.00000 263.00000

186.00000 264.00000

POINTS=5

177.00000 264.00000

176.00000 266.00000

173.00000 267.00000

172.00000 266.00000

171.00000 264.00000

POINTS=5

186.00000 264.00000

184.00000 265.00000

183.00000 265.00000

182.00000 266.00000

181.00000 267.00000

IMAGE=C:\Users\Pc\Desktop\tutte rx in ordine\76.PNG

ID=75

SCALE=0.645077

LM=6

622.00000 735.00000

638.00000 736.00000

634.00000 711.00000

669.00000 692.00000

691.00000 722.00000

672.00000 735.00000

CURVES=3

POINTS=20

641.00000 740.00000

638.00000 734.00000

635.00000 729.00000

632.00000 724.00000

632.00000 717.00000

634.00000 712.00000

636.00000 706.00000

640.00000 701.00000

646.00000 698.00000

651.00000 695.00000

657.00000 694.00000

663.00000 693.00000

669.00000 694.00000

675.00000 696.00000

680.00000 700.00000

685.00000 703.00000

688.00000 709.00000

690.00000 715.00000

691.00000 721.00000

690.00000 727.00000

POINTS=5

642.00000 740.00000

639.00000 746.00000

633.00000 745.00000

628.00000 741.00000

625.00000 735.00000

POINTS=5

691.00000 727.00000

685.00000 729.00000

679.00000 730.00000

673.00000 731.00000

669.00000 736.00000

IMAGE=C:\Users\Pc\Desktop\tutte rx in ordine\77.jpg

ID=76

SCALE=0.141246

LM=6

811.00000 616.00000

825.00000 611.00000

814.00000 591.00000

827.00000 581.00000

859.00000 598.00000

846.00000 615.00000

CURVES=3

POINTS=20

824.00000 612.00000

822.00000 608.00000

820.00000 604.00000

817.00000 601.00000

815.00000 597.00000

814.00000 592.00000

816.00000 589.00000

819.00000 585.00000

823.00000 583.00000

826.00000 581.00000

831.00000 580.00000

835.00000 581.00000

839.00000 582.00000

843.00000 583.00000

847.00000 585.00000

851.00000 587.00000

854.00000 591.00000

857.00000 594.00000

858.00000 598.00000

858.00000 603.00000

POINTS=5

825.00000 613.00000

825.00000 618.00000

821.00000 621.00000

817.00000 620.00000

814.00000 616.00000

POINTS=5

858.00000 603.00000

855.00000 607.00000

850.00000 610.00000

845.00000 612.00000

842.00000 616.00000

IMAGE=C:\Users\Pc\Desktop\tutte rx in ordine\78.jpg

ID=77

SCALE=0.179918
